# CTRP6 is an endogenous complement regulator that can effectively treat induced arthritis

**DOI:** 10.1038/ncomms9483

**Published:** 2015-09-25

**Authors:** Masanori A. Murayama, Shigeru Kakuta, Asuka Inoue, Naoto Umeda, Tomo Yonezawa, Takumi Maruhashi, Koichiro Tateishi, Harumichi Ishigame, Rikio Yabe, Satoshi Ikeda, Akimasa Seno, Hsi-Hua Chi, Yuriko Hashiguchi, Riho Kurata, Takuya Tada, Sachiko Kubo, Nozomi Sato, Yang Liu, Masahira Hattori, Shinobu Saijo, Misao Matsushita, Teizo Fujita, Takayuki Sumida, Yoichiro Iwakura

**Affiliations:** 1 Division of Experimental Animal Immunology, Center for Animal Disease Models, Research Institute for Biomedical Sciences, Tokyo University of Science, Chiba 278-0022, Japan; 2 Laboratory of Molecular Pathogenesis, Center for Experimental Medicine and Systems Biology, The Institute of Medical Science, The University of Tokyo (IMSUT), Tokyo 108-8639, Japan; 3 Department of Computational Biology, Graduate School of Frontier Sciences, The University of Tokyo, Chiba 277-0882, Japan; 4 Core Research for Evolutional Science and Technology (CREST), Japan Science and Technology Agency (JST), Saitama 332-0012, Japan; 5 Department of Internal Medicine, Faculty of Medicine, University of Tsukuba, Tsukuba 305-8575, Japan; 6 Department of Systems Biomedicine, National Research Institute of Child Health and Development, Tokyo 157-8535, Japan; 7 Department of Applied Biochemistry, Tokai University, Hiratsuka, Kanagawa 259-1292, Japan; 8 Department of Molecular Immunology, Medical Mycology Research Center, Chiba University, Chiba 260-8673, Japan; 9 Fukushima Prefectural General Hygiene Institute, Fukushima 960-8142, Japan

## Abstract

The complement system is important for the host defence against infection as well as for the development of inflammatory diseases. Here we show that C1q/TNF-related protein 6 (CTRP6; gene symbol *C1qtnf6*) expression is elevated in mouse rheumatoid arthritis (RA) models. *C1qtnf6*^*−/−*^ mice are highly susceptible to induced arthritis due to enhanced complement activation, whereas *C1qtnf6*-transgenic mice are refractory. The Arthus reaction and the development of experimental autoimmune encephalomyelitis are also enhanced in *C1qtnf6*^−/−^ mice and *C1qtnf6*^−/−^ embryos are semi-lethal. We find that CTRP6 specifically suppresses the alternative pathway of the complement system by competing with factor B for C3(H_2_O) binding. Furthermore, treatment of arthritis-induced mice with intra-articular injection of recombinant human CTRP6 cures the arthritis. CTRP6 is expressed in human synoviocytes, and CTRP6 levels are increased in RA patients. These results indicate that CTRP6 is an endogenous complement regulator and could be used for the treatment of complement-mediated diseases.

## Abstract (web summary)

The complement system contributes to chronic inflammatory diseases. Here the authors show that CRTP6 suppresses the alternative complement pathway and reverses rheumatoid arthritis in a mouse model of the disease.

## Graphical abstract

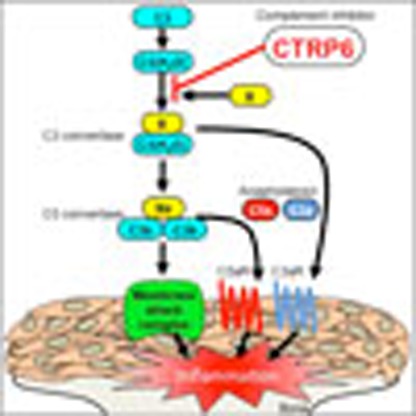

The complement system is a component of the innate immune system, and plays important roles in host defence against microbial infection^1^. The complement system is also suggested to be involved in the pathogenesis of a group of inflammatory diseases. Rheumatoid arthritis (RA) is one of such diseases in which serum concentrations of complement active fragments (C3a and C5a) are elevated^2^,^3^. The development of arthritis is suppressed by the deficiency of the complement system in RA models^4^,^5^,^6^,^7^. The importance of complement activation is also suggested in other inflammatory diseases such as multiple sclerosis (MS) and glomerulonephritis^8^,^9^,^10^.

Complement activation is a proteolytic cascade consisting of three distinct pathways^11^. The classical pathway (CP) is initiated by C1q binding to immune complexes. The lectin pathway (LP) is triggered by binding of mannose-binding lectin or ficolins to pathogen-associated carbohydrate motifs. In the alternative pathway (AP), C3(H_2_O) is initially produced by spontaneous hydrolysis of the internal thioester bond in C3 (Supplementary Fig. 1). Then, factor B binds C3(H_2_O), and the complex is activated by factor D to generate fluid-phase C3 convertase (C3(H_2_O)Bb) (tick-over). On the surface of pathogens, C3(H_2_O)Bb cleaves C3 into C3a and C3b, resulting in C3b deposition. C3b, factor B and factor P form C3 convertase (C3bBbP), which amplifies the reaction (amplification loop). C3b, in combination with Bb, cleaves C5, generating anaphylatoxin C5a and C5b, which form the membrane attack complex (MAC). C3a and C5a have chemotactic and anaphylactic activities; they recruit inflammatory cells to inflammatory sites and activate them to produce inflammatory cytokines^1^,^11^.

RA, an autoimmune disease that attacks multiple joints, affects almost 1% of the world population. Inflammatory cytokines such as tumour necrosis factor (TNF)-α, interleukin (IL)-6, IL-1 and IL-17 play important roles in RA pathogenesis; antibodies and inhibitors against these cytokines are successfully used to treat the disease^12^,^13^. High levels of autoantibodies are a feature of RA, and antibodies against B cells are also effective in treatment of RA^14^.

To identify novel therapeutic targets for the treatment of RA, we analysed gene expressions in two RA models; human T-cell leukaemia virus type-I transgenic (Tg) mice and IL-1 receptor antagonist (IL-1Ra)-deficient (knockout) mice^15^,^16^. We found that the expression of *C1qtnf6* gene, which encodes the C1q/TNF-related protein 6 (CTRP6) protein, was highly enhanced in both models^17^. This gene is also highly expressed in the placenta under physiological conditions^18^. CTRP6 is a glycoprotein of molecular mass of 29 kDa consisting of four domains, these are signal peptide, short N-terminal variable region, collagen domain and C-terminal C1q domain, and is secreted in the serum-forming oligomeric forms^19^. Proteins that contain the C1q domain are classified as members of the C1qTNF family, because the crystal structure of the C1q domain resembles both complement component C1q and TNF-α (ref. 20). The C1q domain of human and mouse CTRP6 are highly homologous (82% amino-acid identity, Supplementary Fig. 2a). CTRP family members have been implicated in host defence, inflammation and glucose metabolism^19^,^21^.

In this study, by generating *C1qtnf6*^−/−^ and Tg mice, we demonstrate that CTRP6 is a novel regulator of the complement AP and plays important roles in the maintenance of pregnancy and the pathogenesis of disease models such as collagen-induced arthritis (CIA)^22^, anti-collagen antibody-induced arthritis (CAIA)^6^, experimental autoimmune encephalomyelitis (EAE)^23^ and Arthus reaction^24^. Because CTRP6 levels are also increased in the serum of RA patients and CTRP6 exerts a potent therapeutic effect on CIA, we suggest that CTRP6 could be used clinically to treat RA and other complement-mediated diseases.

## Results

### Generation of *C1qtnf6* ^−/−^ mice

Because *C1qtnf6* is highly expressed in mouse RA models (Supplementary Fig. 2b), we generated *C1qtnf6*^−/−^ mice to elucidate the pathological roles of CTRP6 in the development of arthritis (Supplementary Fig. 2c–e). *C1qtnf6*^−/−^ mice were fertile and showed no obvious abnormalities before 6 months of age. However, the frequency of obtaining homozygotes from the matings of (129P2/Ola × C57BL/6) F1 background mice was less than the expected Mendelian ratio (wild type (WT):heterozygote:homozygote=159 (31.7%):245 (48.9%):97 (19.4%), *P*=0.021). We used mice after backcrossing with C57BL/6 mice by the high-speed congenic strategy^25^, in which C57BL/6-specific allotypes were confirmed in 84 microsatellite markers, throughout the experiments. Aged *C1qtnf6*^−/−^ mice produced significantly higher levels of autoantibodies, such as antibodies to nuclear antigens (ANAs), IgG- and IgM-type rheumatoid factor (RF) (Supplementary Fig. 3a), although cell populations of lymphoid tissues (Supplementary Fig. 3b–d) and antibody production against thymus-dependent and -independent antigens were normal (Supplementary Fig. 3e–h). Renal function was also normal in these *C1qtnf6*^−/−^ mice (Supplementary Fig. 3i).

### *C1qtnf6* ^−/−^ mice are susceptible to CIA

**Figure 1:**
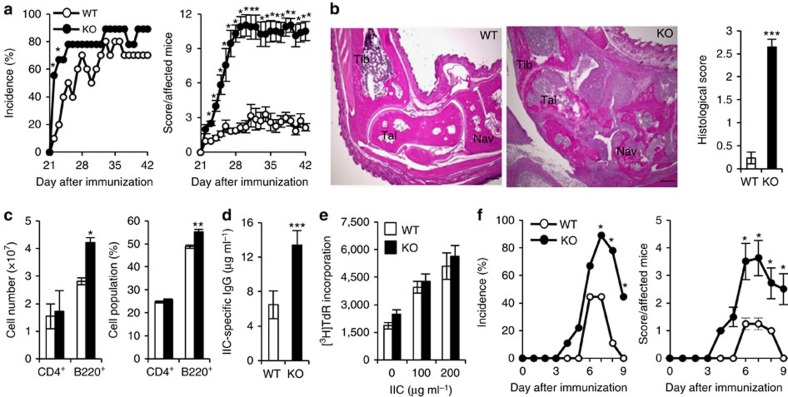
Exacerbation of CIA in *C1qtnf6*^−/−^ mice. (**a**) Incidence (left) and severity score (right) of CIA (WT: *n*=10, knockout (KO): *n*=9). *P<0.05. *χ*^2^-test and Mann–Whitney *U*-test. (**b**) Histopathology (left) and histological score (right) of the ankle joints at day 42 after primary immunization (WT, KO: *n*=9 each). One of representative histologies is shown. Haematoxylin and eosin staining. Tibia, talus and navicular bone are represented as Tib, Tal and Nav, respectively. Bone destruction by pannus formation into talus and navicular bone associated with the presence of osteoclasts and fibroblasts in the KO joint. Scale bar, 300 μm. ***P<0.001. Student's *t*-test. (**c**) At day 42 after immunization, cell number (left) and cell population (right) in inguinal LN cells were analysed by flow cytometry using CD4- and B220-specific antibodies (WT, KO: *n*=8 each). *P<0.05 and **P<0.01. Student's *t*-test. (**d**) The sera at day 42 after primary immunization were collected, and the IIC-specific IgG level was determined by ELISA (WT: *n*=10 and KO: *n*=9). ***P<0.001. Student's *t*-test. (**e**) At 7 days after immunization, inguinal LN cells were cultured with IIC (0, 100, 200 μg ml^−1^). Then IIC-specific proliferative response was measured by [^3^H]TdR incorporation (WT, KO: *n*=8 each). Student's *t*-test. (**f**) Incidence (left) and severity score (right) of CAIA under mild conditions (WT, KO: *n*=9 each). *P<0.05. *χ*^2^-test and Mann–Whitney *U*-test. All data were reproduced in another independent experiment with similar results. Average and s.e.m. are shown.

We next examined the development of CIA in *C1qtnf6*^−/−^ mice. Mice were immunized with chicken type-II collagen (IIC) emulsified in complete Freund's adjuvant (CFA) containing a low concentration of *Mycobacterium tuberculosis* (1.65 mg ml^−1^) to induce mild arthritis. In WT mice under this regimen, the induced arthritis was mild. By contrast, in *C1qtnf6*^−/−^ mice, the onset of arthritis was earlier, and the severity score was higher than in WT mice, although the incidence of arthritis was similar between the two strains (Fig. 1a). Histology of the joints of WT mice revealed mild pathological changes, whereas that of *C1qtnf6*^−/−^ joints revealed much more severe changes, including proliferation of synovial lining cells, infiltration of inflammatory cells and bone destruction associated with pannus formation (Fig. 1b). The number and percentage of CD4^+^ T cells in lymph node (LN) were similar between *C1qtnf6*^−/−^ and WT mice, whereas the B220^+^ B-cell population was significantly expanded in *C1qtnf6*^−/−^ mice (Fig. 1c). Moreover, IIC-specific IgG levels after IIC immunization were significantly higher in *C1qtnf6*^−/−^ than in WT mice (Fig. 1d). The proliferative response to IIC of LN cells in *C1qtnf6*^−/−^ mice was similar to that of WT mice, suggesting that T-cell priming was normal (Fig. 1e). These results suggest that *C1qtnf6*^−/−^ mice are more susceptible to CIA than WT mice.

We also analysed susceptibility of *C1qtnf6*^−/−^ mice to CAIA^6^, in which antibodies against IIC were directly injected to induce arthritis. We found that *C1qtnf6*^−/−^ mice were much susceptible to CAIA than WT mice (Fig. 1f). Because CAIA is independent from antibody production, these results suggest that the effect of CTRP6 on the autoantibody-induced inflammation is important rather than on the production of antibodies.

### *C1qtnf6* Tg mice are refractory to the development of CIA

Next, we generated *C1qtnf6* Tg mice carrying mouse *C1qtnf6* under the control of the CAG promoter^26^ to examine the effects of excess CTRP6. Serum CTRP6 levels were ∼2.5 times higher in *C1qtnf6* Tg mice than in non-Tg littermates (Supplementary Fig. 4a–d). Heterozygous *C1qtnf6* Tg mice were born in the expected Mendelian ratio (WT:*C1qtnf6* Tg=290 (53.5%):252 (46.5%), *P*=0.273), were fertile and showed no obvious abnormalities including the renal function (Supplementary Fig. 4e).

**Figure 2:**
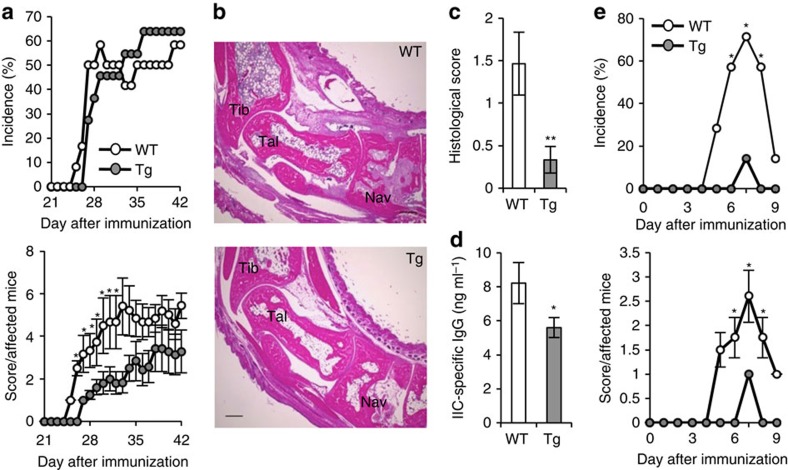
Amelioration of CIA in *C1qtnf6* Tg mice. (**a**) Incidence (upper) and severity score (lower) of CIA (WT: *n*=12, Tg: *n*=11). *P<0.05. *χ*^2^-test and Mann–Whitney *U*-test. (**b**,**c**) Histopathology (**b**) and histological score (**c**) of the ankle joints at day 42 after primary immunization (WT, Tg: *n*=9 each). Haematoxylin and eosin staining. Tibia, talus and navicular bone are represented as Tib, Tal and Nav, respectively. Bone destruction by pannus formation into navicular bone associated with the presence of osteoclasts and fibroblasts is seen in the WT joint. Scale bar, 300 μm. One of representative histologies is shown. **P<0.01. Student's *t*-test. (**d**) IIC-specific IgG levels at day 42 after primary immunization (WT: *n*=12, Tg: *n*=11). *P<0.05. Student's *t*-test. These results were reproduced in another independent experiment with similar results. (**e**) Incidence (upper) and severity score (lower) of CAIA under severe conditions. These data were combined from two independent experiments (WT, Tg: *n*=7 each). *P<0.05. *χ*^2^-test and Mann–Whitney *U*-test. Average and s.e.m. are shown.

We examined the susceptibility of *C1qtnf6* Tg mice to CIA, using a higher concentration of *M. tuberculosis* (2.5 mg ml^−1^) in CFA to induce severe arthritis. The arthritic severity score was significantly lower in *C1qtnf6* Tg mice than in WT mice, although the incidence was similar (Fig. 2a). Histology of the joints of *C1qtnf6* Tg mice exhibited milder pathological changes than that of WT mice (Fig. 2b,c). Serum IIC-specific IgG levels were also decreased in these *C1qtnf6* Tg mice (Fig. 2d). Furthermore, *C1qtnf6* Tg mice were refractory against the induction of CAIA compared with WT mice (Fig. 2e). These results suggest that high concentration of CTRP6 can suppress the development of arthritis.

### CTRP6 specifically inhibits complement AP

**Figure 3:**
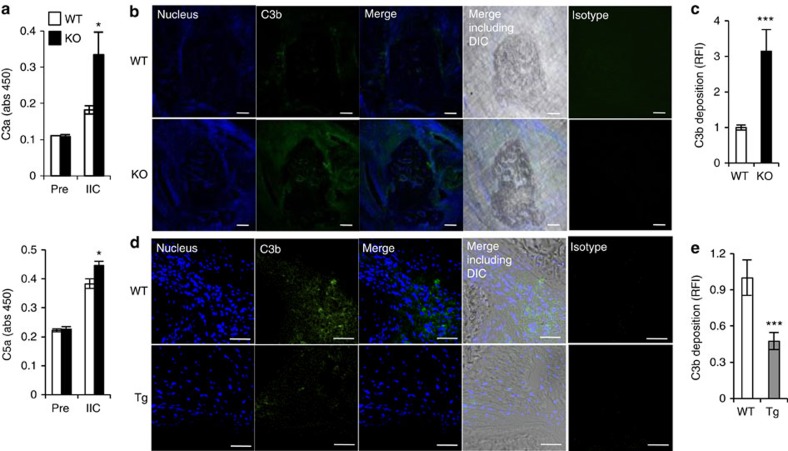
Complement activation in CIA-induced mice. (**a**) Plasma samples before (pre) and 7 days after (IIC) IIC immunization were collected, and C3a (upper) and C5a (lower) levels were measured by ELISA (WT, KO: *n*=8 each). *P<0.05. Student's *t*-test. The data were reproduced in another independent experiment with similar results. (**b**,**c**) Cryostat sections of the WT and *C1qtnf6*^−/−^ ankle joints at day 42 after primary immunization were stained with anti-C3b antibody and 4′,6-diamidino-2-phenylindole (DAPI) (nucleus). The photomicrographs were taken with fluorescence and differential interference contrast (DIC) optics. Scale bar, 20 μm. One of representative histologies is shown. The relative fluorescence intensity (RFI) of C3b (**c**) was determined by ImageJ (WT, KO: *n*=6 each). ***P<0.001. Student's *t*-test. (**d**,**e**) Cryostat sections of WT and *C1qtnf6* Tg ankle joints at day 42 after primary immunization were stained with anti-C3b antibody and DAPI (nucleus). The photomicrographs were taken with fluorescence and DIC optics. Scale bar, 50 μm. One of representative histologies is shown. The relative fluorescence intensity of C3b (**e**) was determined by ImageJ (WT, Tg: *n*=7 each). ***P<0.001. Student's *t*-test. Average and s.e.m. are shown.

Interestingly, we found that the concentrations of C3a and C5a in the plasma of IIC-immunized mice were increased in *C1qtnf6*^−/−^ mice compared with WT mice (Fig. 3a). Furthermore, the deposition of C3b in the joints was increased in *C1qtnf6*^−/−^ mice (Fig. 3b,c). In contrast, the deposition of C3b in the joints was lower in *C1qtnf6* Tg mice than in WT mice (Fig. 3d,e). These results suggest that CTRP6 regulates complement activation.

**Figure 4:**
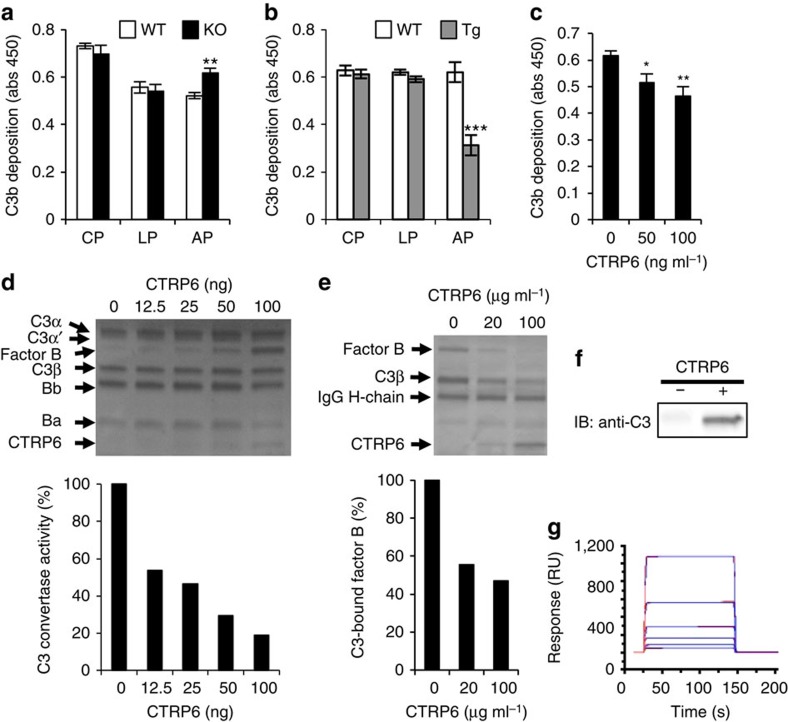
CTRP6 specifically regulates AP activation. (**a**–**c**) Complement activation was determined by C3b deposition. (**a**) The CP, LP and AP in 10% WT and *C1qtnf6*^−/−^ mice serum were activated by the OVA/anti-OVA immune complex, mannans and LPS, respectively (WT, KO: *n*=8 each). *P<0.05, **P<0.01 and ***P<0.001. Student's *t*-test. (**b**) Complement activation in 10% WT and *C1qtnf6* Tg mouse serum (WT, Tg: *n*=8 each). (**c**) The AP in 10% *C1qtnf6*^−/−^ serum was activated by LPS in the presence of rhCTRP6 (*n*=8). (**d**) AP C3 convertase activity. RhCTRP6 was incubated with a mixture of human C3, factor B and factor D. The reaction mixtures were subjected to SDS–PAGE and were visualized by CBB staining (upper). AP C3 convertase activity (lower) was calculated by the intensity of the Bb protein band. Images have been cropped for presentation. Full-size images are presented in Supplementary Fig. 5g. (**e**) After incubation of human C3(H_2_O) with human factor B and rhCTRP6, C3 was immunoprecipitated with anti-C3 antibody, and immunoprecipitates were subjected to SDS–PAGE. After staining with CBB (upper), the factor B band intensity (lower) was quantified. Images have been cropped for presentation. Full-size images are presented in Supplementary Fig. 5h. (**f**) RhCTRP6 (+) or dilution buffer (−) was added to *C1qtnf6*^−/−^ serum, and rhCTRP6-associated proteins were immunoprecipitated with anti-CTRP6 antibody. Immunoprecipitates were subjected to SDS–PAGE, and C3 and CTRP6 were detected with anti-C3 and anti-CTRP6 antibody, respectively. Images have been cropped for presentation. Full-size images are presented in Supplementary Fig. 5i,j. (**g**) C3–CTRP6 interaction was detected by surface plasmon resonance analysis. Human C3 (0, 15.625, 31.25, 62.5, 250 and 500 nM) was flowed over a rhCTRP6-immobilized sensor chip. The response is expressed in resonance response units (RUs). All data were reproduced in another independent experiment. Average and s.e.m. are shown.

Then, we examined the effects of CTRP6 deficiency on three complement pathways *in vitro*. Plates coated with the ovalbumin (OVA)/anti-OVA immune complex, mannan and lipopolysaccharide (LPS) were used to activate the CP, LP and AP, respectively. Sera were diluted with GVB^++^ buffer containing Ca^2+^ for CP and LP assays, while sera for determination of AP activity were diluted with GVB/Mg^2+^EGTA to eliminate Ca^2+^, because the AP is Ca^2+^ independent. We found that AP activity was specifically enhanced in *C1qtnf6*^−/−^ sera compared with WT sera (Fig. 4a). Furthermore, MAC formation under AP activation conditions was enhanced in *C1qtnf6*^−/−^ mouse sera (Supplementary Fig. 5a). In contrast, *C1qtnf6* Tg mouse serum specifically suppressed AP activation (Fig. 4b; Supplementary Fig. 5b). C3 and factor B levels were comparable in WT, *C1qtnf6*^−/−^ and *C1qtnf6* Tg mouse sera (Supplementary Fig. 5c). These results suggest that CTRP6 specifically suppresses AP activation.

Then, we directly analysed the effect of CTRP6 on AP activation *in vitro*. Because C1q domain of human and mouse CTRP6 are highly homologous and CTRP6 mainly forms oligomers in the serum (Supplementary Fig. 5d), we analysed the effect of monomeric exogenous recombinant human CTRP6 (rhCTRP6) on the complement activity of mouse sera. *C1qtnf6*^−/−^ sera were used to avoid possible influence of endogenous CTRP6. Results showed that rhCTRP6 specifically inhibited AP activation in a dose-dependent manner (Fig. 4c; Supplementary Fig. 5f).

On the basis of these findings, we investigated in further detail the effects of CTRP6 on AP activation. Upon activation of the AP, spontaneously generated C3(H_2_O) forms a complex with factor B, followed by cleavage of factor B by factor D to generate C3(H_2_O)Bb, a C3 convertase, and Ba. C3 is composed of C3α and C3β; C3 is cleaved to C3a and C3b (consisting of C3α′ and C3β) by C3 convertase (Supplementary Fig. 6)^27^. After incubation of human C3, factor B and factor D in the presence of various concentrations of rhCTRP6 *in vitro*, the reaction mixture was subjected to SDS–polyacrylamide gel electrophoresis (PAGE) and visualized by Coomassie brilliant blue (CBB) staining (Fig. 4d; Supplementary Fig. 5g). The result showed that rhCTRP6 inhibited proteolytic activation of factor B in a dose-dependent manner, indicating that CTRP6 suppressed C3 convertase formation. Next, we examined the effect of CTRP6 on the formation of the C3(H_2_O)–factor B complex, the first step of C3 tick-over. After incubating a mixture of human C3(H_2_O), human factor B and rhCTRP6, C3-containing protein complexes were immunoprecipitated using anti-C3 antibodies. Co-immunoprecipitation of factor B decreased as a function of rhCTRP6 dose (Fig. 4e; Supplementary Fig. 5h), suggesting that CTRP6 competitively inhibits factor B binding to C3(H_2_O). Furthermore, exogenously added rhCTRP6 co-precipitated with C3 from *C1qtnf6*^−/−^ serum (Fig. 4f; Supplementary Fig. 5i,j). Direct binding of rhCTRP6 to human C3(H_2_O), but not to C3b, was shown by surface plasmon resonance (SPR) analysis (*K*_D_=1.1 × 10^−6^) (Fig. 4g; Supplementary Fig. 5k). Furthermore, we examined the association between CTRP6 and factor H, because factor H also binds C3(H_2_O)^28^ and adiponectin, one of the CTRP family associated with factor H to modulate the AP activation^29^. However, CTRP6 did not bind factor H nor influence its activity (Supplementary Fig. 5l,m). These results demonstrated that CTRP6 regulates AP activation by binding to C3(H_2_O).

### *C1qtnf6* deficiency enhances the Arthus reaction and EAE

The Arthus reaction is an immune complex-mediated type-III hypersensitivity that depends on complement activation^24^. To confirm that CTRP6 is a complement inhibitor, we examined the sensitivity of *C1qtnf6*^−/−^ mice to an IgG-mediated reverse passive Arthus (RPA) reaction. We found that vascular permeability was significantly enhanced in *C1qtnf6*^−/−^ mice compared with WT mice (Supplementary Fig. 7a). These results support the notion that CTRP6 is a regulator of the complement system.

MS is an autoimmune disease of the central nervous system. Studies of EAE, an experimental model of MS, have suggested that the AP, but not the CP or LP, is important for the pathogenesis^30^,^31^. Another recent study has also suggested the involvement of complement activation in MS pathogenesis^8^. Therefore, we investigated whether *C1qtnf6* deficiency affected the development of EAE. The severity score was much higher in *C1qtnf6*^−/−^ mice than in WT mice, although the incidence of disease was similar between the two strains (Supplementary Fig. 7b). These results provide further support for the notion that CTRP6 is a novel regulator of the complement system.

### CTRP6 is effective for the treatment of CIA

**Figure 5:**
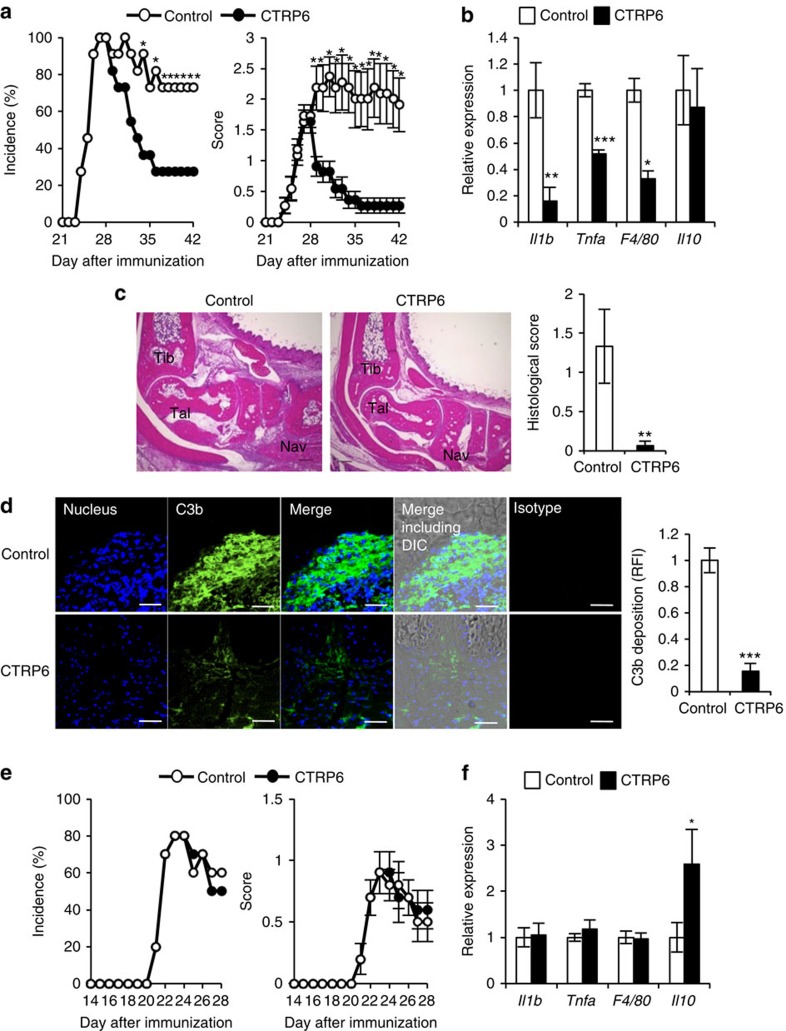
Treatment of CIA with rhCTRP6. (**a**,**b**) RhCTRP6 or PBS as a control was daily injected into the articular cavity of the left (rhCTRP6) or right (rhCTRP9) knee joint of CIA-induced DBA/1J mice from day 28 (*n*=11). (**a**) Incidence (left) and severity score (right) of CIA. *P<0.05. *χ*^2^-test and Mann–Whitney *U*-test. (**b**) Messenger RNA expression in the joints was measured by semi-quantitative PCR analysis. *P<0.05, **P<0.01, and ***P<0.001. Student's *t*-test. (**c**) Histology (left) and histological score (right) of the ankle joints at day 42. Haematoxylin and eosin. Tibia, talus and navicular are represented as Tib, Tal and Nav, respectively. Scale bar, 300 μm. One of representative histologies is shown (*n*=6). **P<0.01. Student's *t*-test. (**d**) Cryostat sections of the joints were stained with anti-C3b antibody, isotype IgG and 4′,6-diamidino-2-phenylindole (nucleus) (left). One of representative histologies is shown. The photomicrographs were taken with fluorescence and differential interference contrast (DIC) optics. The relative fluorescence intensity of C3b (right) was determined by ImageJ (*n*=4). Scale bar, 50 μm. ***P<0.001. Student's *t*-test. (**e**,**f**) RhCTRP6 or rhCTRP9 as a control was daily injected into the articular cavity of the left (rhCTRP6) or right (rhCTRP9) knee joint of CIA-induced *C3*^−/−^ mice from day 21 (*n*=10). (**e**) Incidence (left) and severity score (right) of CIA in *C3*^−/−^ mice. *χ*^2^-test and Mann–Whitney *U*-test. (**f**) Messenger RNA expression in the joints of *C3*^*−/−*^ mice after treatment with rhCTRP6 are shown relative to those of control-treated mice were measured by semi-quantitative PCR analysis. *P<0.05. Student's *t*-test. Average and s.e.m. are shown.

Next, we examined whether CTRP6 can treat established arthritis. We immunized DBA/1J mice with IIC/CFA (*M. tuberculosis* 0.5 mg ml^−1^), followed by a booster injection with IIC/CFA on day 21. Starting on day 28, we performed daily injections of rhCTRP6 into the articular cavity of the knee joints of affected mice (rhCTRP6 into left leg; PBS or rhCTRP9 into right as a control), and evaluated the swelling of the ankle joints. As shown in Fig. 5a and Supplementary Fig. 8, ankle joint swelling was promptly ameliorated by the injection of rhCTRP6 to the knee joint, whereas the ankle joints of the PBS- or rhCTRP9-injected side still remained swollen. In the rhCTRP6-treated joints of the WT mice, messenger RNA (mRNA) expression levels of *Il1b*, *Tnfa* and *F4/80* were decreased, whereas *Il10* mRNA level did not change (Fig. 5b). Histology of joints revealed that PBS-injected joints were destroyed, but rhCTRP6-injected joints were normal (Fig. 5c). C3b deposition was decreased in the rhCTRP6-injected joints relative to the PBS-injected joints (Fig. 5d). Next, we injected rhCTRP6 to arthritic *C3*^−/−^ mice to investigate whether CTRP6 can treat arthritis in a complement-independent fashion. In contrast, rhCTRP6 failed to treat arthritis in *C3*^−/−^ mice (Fig. 5e), suggesting that C3 is involved in the manifestation of CTRP6 action. Messenger RNA expression levels of *Il1b*, *Tnfa* and *F4/80* were not changed in these rhCTRP6-treated *C3*^−/−^ mice. However, *Il10* mRNA levels were increased (Fig. 5f). These results suggest that CTRP6 is effective for the treatment of autoimmune arthritis.

### CTRP6 is expressed in synovial cells of RA patients

**Figure 6:**
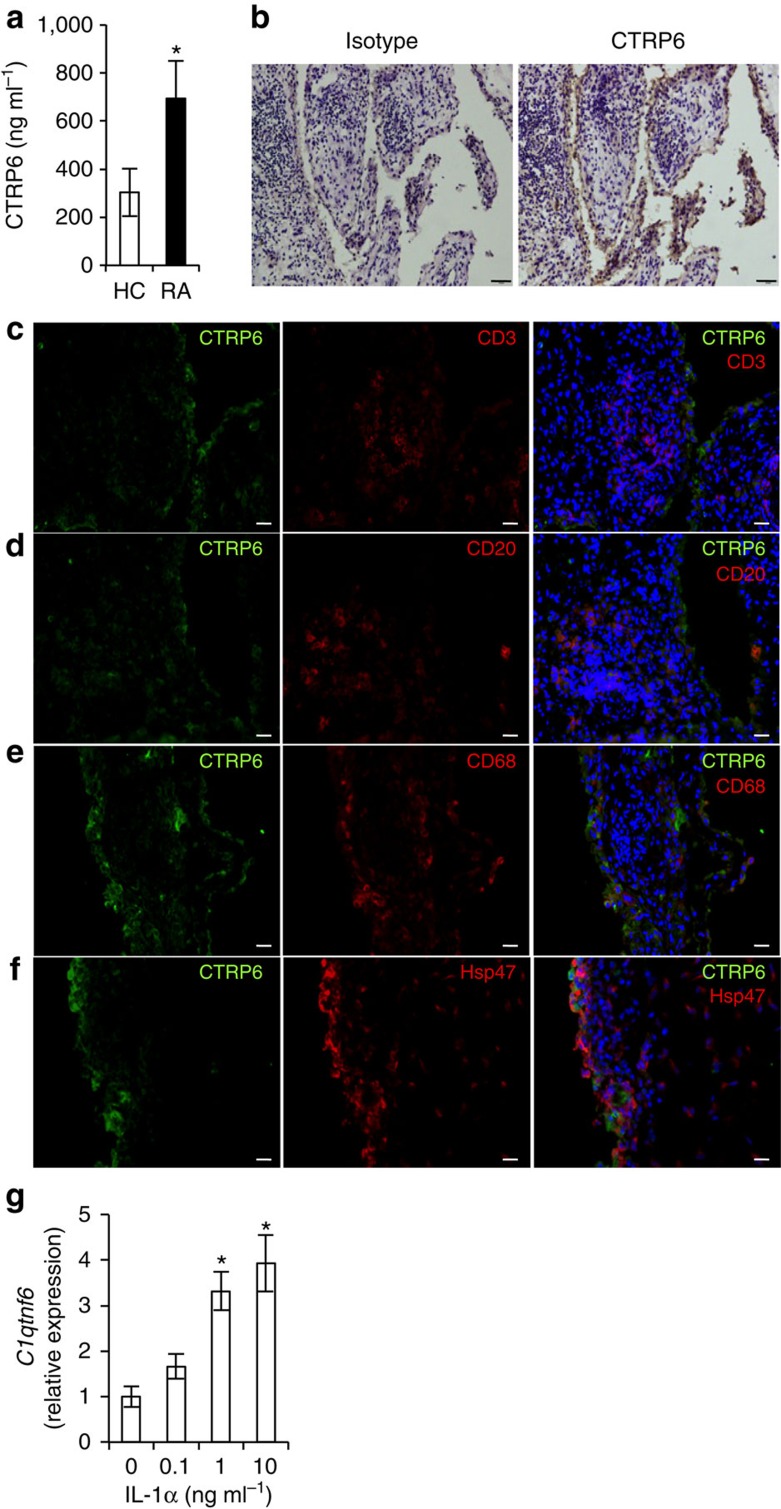
CTRP6 is expressed in synoviocytes of arthritic joints of RA patients. (**a**) The CTRP6 levels in the serum of healthy controls (HC) and RA patients (RA) were measured by ELISA (HC: *n*=22, RA *n*=30). *P<0.05. Student's *t*-test. (**b**) Synovium from a RA patient was stained with anti-CTRP6 antibody or isotype IgG using diaminobenzidine tetrahydrochloride staining. Scale bar, 50 μm. (**c**–**f**) Cryostat sections of synovia from RA patients were stained with anti-CTRP6 antibody and 4′,6-diamidino-2-phenylindole (nucleus), and either anti-CD3 antibody (**c**), or anti-CD20 antibody (**d**), or anti-CD68 antibody (**e**), or anti-Hsp47 antibody (**f**). Scale bar, 20 μm. (**g**) Expression of *C1qtnf6* in primary fibroblast-like synoviocytes stimulated with IL-1α (*n*=3) is shown. *P<0.05. Student's *t*-test. Average and s.e.m. are shown.

Finally, we investigated CTRP6 expression in RA patients. We found that CTRP6 concentration was increased in sera of RA patients compared with that of healthy controls (Fig. 6a). CTRP6 was detected in synovial lining cells of RA patients (Fig. 6b). Double-immunostaining using RA synovial tissues showed that CTRP6 expression was localized to Hsp47^+^ synovial cells^32^ (Fig. 6c–f; Supplementary Fig. 9). Furthermore, we found that *C1qtnf6* expression in primary fibroblast-like synoviocytes was enhanced by IL-1α stimulation (Fig. 6g). These results indicate that CTRP6 expression is elevated in synovial cells in RA patients.

## Discussion

Because C3a and C5a have chemotactic, anaphylatoxic and IgG production-promoting activity, and the MAC has strong cytolytic activity, complement activity must be strictly controlled under physiological conditions. When this control fails, excess or inappropriate activation of a complement pathway can cause multiple human diseases^1^,^8^,^33^. Thus, complement regulatory factors are important for the homeostasis of the complement system. In this report, we have shown that CTRP6 is a novel endogenous regulator of the AP and the AP was excessively activated in *C1qtnf6*^*−/−*^ mice. CTRP6 efficiently suppresses the AP ‘tick-over' by inhibiting factor B binding to C3(H_2_O) in a competitive manner. It is known that AP activation is also regulated by factor I and factor H; both regulate the ‘AP amplification loop' (Supplementary Fig. 1), and factor H also blocks the tick-over reaction. Factor I, in cooperation with factor H, cleaves C3b to generate inactive C3b (iC3b). Factor H also accelerates decay of C3 convertase (C3bBb) by dissociating Bb^34^. Factor H-deficient (*Cfh*^−/−^) mice spontaneously develop membrane-proliferative glomerulonephritis due to C3 dysregulation, but factor I-deficient (*Cfi*^−/−^) mice do not develop glomerulonephritis, in spite of C3 dysregulation^9^,^10^. C1-inhibitor-deficient (*C1inh*^*−/−*^) mice show increased vascular permeability^35^. Furthermore, deficient mice of the complement receptor 1-related gene/protein-y (*Crry*) gene, which regulates CP and AP activation, are 100% embryonic lethal due to spontaneous complement activation^36^.

It is reported that factor H also interacts with C3(H_2_O) via the residues Glu-744 and Glu-747 of C3 (ref. 37). However, factor B–C3(H_2_O) interaction, which is inhibited by CTRP6, occurs at different residues from the factor-H-binding site^38^. Furthermore, CTRP6 did not bind factor H nor influence its activity (Supplementary Fig. 5l,m). Thus, the inhibitory mechanism of CTRP6 is different from that of factor H. Although the binding affinity between C3(H_2_O) and CTRP6 was relatively low (*K*_D_=1.1 × 10^−6^), the real binding affinity may be greater than this because CTRP6 forms oligomers in the serum (Supplementary Fig. 5d). Because the binding of factor B and C3(H_2_O) initiates AP activation, the regulation of this step should be critically important for the AP activation. Actually, we found that the frequency of homozygotes from the mating between *C1qtnf6* F1 heterozygotes on the (129P2/Ola × C57BL/6) background was less than that expected by the Mendelian ratio, partially resembling *Crry*^−/−^ mice^36^. This observation suggests a beneficial role of CTRP6 in the development of fetus, consistent with the high levels of expression in the placenta. Furthermore, aged *C1qtnf6*^−/−^ mice produced significantly higher levels of autoantibodies, probably reflecting the increase of C3a and C5a, which can promote IgG production^1^, although we did not observe any renal failure caused by immune complexes. Thus, CTRP6 plays important roles in the regulation of the complement system under the physiological conditions. Although adiponectin, another CTRP family member, also suppresses the activation of the AP, this occurs through the activation of factor H^29^, a different mechanism from CTRP6.

AP activation is implicated in the development of RA and other inflammatory diseases^5^,^6^,^7^,^8^,^39^. IgGs in immune complexes with agalactosylated complex type *N*-glycans, which are increased in sera of RA patients^40^ as well as in RA models^41^, can activate the AP^42^. In this report, we showed that the development of CIA, in which complement activation plays a deleterious role^39^, was greatly exacerbated in *C1qtnf6*^−/−^ mice, and ameliorated in *C1qtnf6* Tg mice. These findings suggest that CTRP6 is involved in the regulation of arthritis development by suppressing the AP. Although we observed changes of anti-IIC levels in *C1qtnf6*^−/−^ mice and *C1qtnf6* Tg mice upon induction of CIA, these are secondary effects of AP activation/suppression and are not responsible for their severity score changes, because the severity scores in CAIA model, in which antibody production is not necessary, also changed in *C1qtnf6*^−/−^ mice and in *C1qtnf6* Tg mice.

The involvement of the AP in the development of RA was also suggested in other RA models. The development of arthritis in K/BxN serum-transferred mice, a model in which autoantibodies against glucose-6-phosphate isomerase play an important role, is suppressed by the deficiency of factor B, C3, C5 or C5a, but not by C1q or MBP-A, suggesting the involvement of the AP in the pathogenesis^5^. The development of arthritis in SKG mice, which carry a mutation in the T-cell receptor component ZAP70, is suppressed by the deficiency of C5aR^7^. In that report, the authors showed that C5a signalling induces the development of Th17 cells through induction of cytokines such as IL-6, IL-1β, IL-23 and granulocyte–macrophage colony-stimulating factor in macrophages^7^. Because IL-17 can enhance antibody production^43^, the promotion of Th17 cell differentiation also explains why much higher levels of anti-IIC IgG were produced in *C1qtnf6*^−/−^ mice than WT mice upon immunization with IIC. Furthermore, it was recently reported that absence of C3a and C5a signalling can promote Treg cell differentiation by inducing TGF-β from T cells^44^, implying that excess AP activation may suppress the development of Treg cells. Therefore, excess activation of the complement system not only directly induces inflammation by recruiting macrophages and neutrophils but also induces autoimmunity and inflammation by enhancing Th17 cell differentiation, suppressing Treg cell differentiation and enhancing antibody production. Thus, CTRP6 may be an important regulator of inflammation by preventing excess complement activation under pathological conditions.

We have shown that CTRP6 injection is effective for the treatment of CIA. When rhCTRP6 was injected into the articular cavity of the left knee, clear suppressive effect on the ankle swelling of the same leg was observed, indicating that knee-joint-injected rhCTRP6 diffuses into the ipsilateral ankle joint. We used a contralateral leg as the control, which was injected with PBS or CTRP9 to the knee joint. Although the effect of rhCTRP6 to the contralateral leg was only marginal, the severity score of the control ankle joints could be lower than those of rhCTRP6-untreated mice due to the possible contribution of systemic effects. *C3*^*−/−*^ mice were refractory against the CTRP6 treatment, indicating that the inhibition of the AP is responsible for the therapeutic effect. We observed that rhCTRP6 enhanced IL-10 production from WT and *C1qtnf6*^−/−^ bone marrow (BM)-derived macrophages (Supplementary Fig. 10), consistent with a report that shows IL-10 induction by CTRP6 from Raw264.7 macrophages^45^. Importantly, *Il10* mRNA expression was still elevated in the CTRP6-treated joints of *C3*^−/−^ mice (Fig. 5e). These observations suggest that the therapeutic effect of CTRP6 on CIA mainly depends on its complement inhibitory activity, but not on the IL-10-inducing activity. In support for this notion, it was reported that the disease activity of RA and circulating levels of IL-10 are not correlated^46^.

CTRP family including adiponectin is known as an adipokine. The CTRP family regulates systemic energy homeostasis via adipoR1, adipoR2 and unknown receptors^47^. And we have already reported that CTRP3 is important for the development of autoimmune disease, but is not a complement regulator^19^,^21^. Thus, it remains a possibility that CTRP6 has complement-independent functions.

Suppression of the complement system is an attractive strategy for the treatment of inflammatory diseases involving excess complement activation. Indeed, an antibody against C5a (eculizumab) has been successfully used for the treatment of paroxysmal nocturnal hemoglobinuria^48^. The involvement of the complement system is also suggested in other diseases. We have shown that EAE, in which the involvement of the AP has been implicated^8^, is significantly enhanced in *C1qtnf6*^−/−^ mice. Furthermore, *C1QTNF6* locus is a susceptibility locus associated with autoimmune diseases including RA and type-1 diabetes^49^,^50^,^51^. Together, these observations suggest that CTRP6 may play important roles not only in RA but also in other inflammatory diseases that involve AP activation, such as MS, type-1 diabetes^52^, age-related macular degeneration, systemic lupus erythematosus and glomerulonephritis^1^,^8^,^53^. Thus, CTRP6 is a promising candidate of the medicine for the treatment of RA and other inflammatory diseases in humans.

## Methods

### Reverse transcription and real-time PCR

Total RNA was extracted using Sepasol-RNA I Super (Nacalai Tesque, Japan). RNA was reverse transcribed using the High-Capacity cDNA Reverse Transcription Kit (Applied Biosystems, USA). Reverse transcription (RT)-PCR using Taq (TaKaRa, Japan) and iCycler System (Bio-Rad, USA) was performed with the sets of primers described in Supplementary Table 1. Quantitative real-time RT-PCRs were performed using SYBR Premix Ex Taq (TaKaRa) and an iCycler System (Bio-Rad) with the sets of primers described in Supplementary Table 1.

### Mice

*C1qtnf6*^−/−^ mice were generated using E14.1 embryonic stem (ES) cells, and *C1qtnf6* Tg mice were generated by pronuclear microinjection into C57BL/6J mouse embryos and were used as heterozygotes as described in the following sections. *C3*^−/−^ mice (C57BL6/J background) were obtained from Jackson laboratories. Mice were kept under specific pathogen-free conditions in the clean rooms at the Center for Experimental Medicine and Systems Biology, Institute of Medical Science, University of Tokyo, and the Research Institute for Biomedical Sciences, Tokyo University of Science. All experiments were approved by the committee of Life Science Research Ethics and Safety of the University of Tokyo and the Animal Care and Use Committee of Tokyo University of Science, and were conducted according to the institutional ethical guidelines for animal experiments and safety guidelines for gene manipulation experiments.

### Generation of *C1qtnf6* ^−/−^ mice

The *C1qtnf6*^−/−^ allele was created by homologous recombination in E14.1 ES cells. Homology arms of the *C1qtnf6* gene-targeting vector were amplified from genomic DNA of E14.1 ES cell by PCR methods using the primer set 1 and 2 for the 5′ arm and 3′ arm, respectively (Supplementary Table 2). The lengths of the 5′ and 3′ arms are 3.5 and 6.9 kb, respectively. The targeting vector was constructed by replacing the genomic locus containing the third exon of the *C1qtnf6* gene, which encodes the C1q domain, with a neomycin-resistance gene (*Neo*^*r*^) under the control of a PGK1 promoter for positive selection. A diphtheria toxin A gene under the MC1 promoter was ligated to the 3′ end of the targeting vector for negative selection. In addition, the stop codon and HindIII sequence for screening were introduced into exon 2 in the 5′ homology arm of the targeting vector. The targeting vector was electoroporated into ES cells and selected in G418 (Nacalai Tesque). We screened 223 neomycin-resistant ES clones by PCR and Southern blotting. The first screening was performed by PCR using the primer set 3 (Supplementary Table 2). For positive clones from the PCR screening, we confirmed correct homologous recombination by Southern blotting using 5′ and 3′ flanking probes. Genomic DNA of the targeting clones was digested using HindIII and EcoRV, respectively, for detection by the 5′ and 3′ probe. The DNA fragment for the 5′ probe was amplified using the primer set 4 and 5 for 5′ and 3′ probe, respectively (Supplementary Table 2). As a result of this screening, we obtained one targeted clone (clone ID: 3C2) (targeting efficiency: 0.5%). Using this clone, chimeric mice were generated by an aggregation method modified from previous reports^54^,^55^. The contribution of ES cells to chimeric mice was checked by coat colour. To generate heterozygous offspring, male chimeric mice were mated with C57BL/6J female mice, and (129P2/Ola × C57BL/6) F1 progeny were obtained. In this report, we used mice of the C57BL/6 background. Mice were backcrossed with C57BL/6J mice for five generations using the high-speed congenic strategy^25^. The proportion of C57BL/6J homozygous loci in male mice of the N5 generation was 100% (84 markers). The genotyping of *C1qtnf6*^−/−^ mice were carried out using the following primers: WT sense primer 1, mutant sense primer 1 and common antisense primer 1 (Supplementary Table 3). The common primer and the WT primer were used to detect the WT allele (686 bp), and the common primer and mutant primer were used to detect the mutant allele (454 bp).

### Generation of *C1qtnf6* Tg mice

An EcoRI-flanked full-length mouse *C1qtnf6* complemntary DNA fragment was obtained by RT-PCR using the primer set 6 (Supplementary Table 2). This fragment was introduced into the EcoRI site of the pCXN2 plasmid^26^. The transgene was designed for ectopic expression of *C1qtnf6* under control of the CAG promoter. We confirmed by direct sequencing that the fusion gene contained no errors. CAG-*C1qtnf6*-polyA fragments were digested by BamHI and PvuI to eliminate the plasmid backbone sequence. A purified CAG-*C1qtnf6*-polyA fragment was microinjected into the pronuclei of fertilized eggs of C57BL/6J mice (CLEA Japan, Japan). Viable embryos were transferred into the oviducts of pseudopregnant female ICR mice (Japan SLC, Japan), and pups were obtained. Mice carrying the CAG-*C1qtnf6* transgene were identified by PCR screening using the primer set 7 (Supplementary Table 2). Then, we confirmed Tg founder mice by Southern blot analysis of tail DNA, using a probe for the HindIII/EcoRI site in the *C1qtnf6* sequence. The Tg probe was amplified by PCR using the primer set 8 (Supplementary Table 2). The genotyping of *C1qtnf6* Tg mice was carried out using the following primers: WT sense primer 2, mutant sense primer 2 and common antisense primer 2 (Supplementary Table 3). The Tg mice were used as heterozygotes.

### Titration of CTRP6

To measure CTRP6 levels in serum, we collected mouse sera and diluted 10 times with PBS (pH 7.4). Plates (Nunc, Denmark) were coated with polyclonal rabbit antibodies against human and mouse CTRP6 (AnaSpec, USA, 54561, 1:1,000 dilution). After incubation with diluted serum (50 μl) for 60 min, biotin-labelled polyclonal rabbit antibodies against human and mouse CTRP6 (AnaSpec, 54562, 1:1,000 dilution) were added, and the absorbance at 450 nm was inspected after incubation with avidin-conjugated horseradish peroxidase (HRP). RhCTRP6 (BioVendor, Czech Republic) was used as the standard.

### Titration of autoantibodies

We collected serum from aged mice (male 129P2_Ola × C57BL/6J F1 background at 1 year of age) and titrated autoantibodies, such as ANA and RF. To measure ANA, we performed enzyme-linked immunosorbent assay (ELISA) with plates coated with nuclear antigens and HRP-conjugated polyclonal goat antibodies to mouse IgG using the Autoimmune ELISA kit (Alpha Diagnostic, USA, 5210). To measure RF, we performed ELISA with plates coated with heat-denatured rabbit IgG (Santa Cruz, USA, sc-2027, 1:8 dilution) and alkaline phosphatase-conjugated polyclonal rabbit antibodies to mouse IgG (Zymed, USA, 62–6622, 1:1,000 dilution) and IgM (Santa Cruz, sc-2070, 1:1,000 dilution).

### Flow cytometric analysis

For flow cytometry, we harvested thymus, spleen and LN from mice (male 129P2_Ola × C57BL/6J F1 background at 8 weeks of age) and stained cells with a 100-fold dilution of phycoerythrin-Cy7-, fluorescein isothiocyanate (FITC)-, APC-Cy7-, Pacific-Blue-, APC- and phycoerythrin-conjugated monoclonal antibodies (mAbs). We purchased rat or hamster monoclonal antibodies to mouse CD4 (GK1.5), CD3 (145-2C11), B220 (RA3–6B2) and CD11b (M1/70) from Biolegend (USA) and CD8 (53-6.7) from eBioscience (USA) and CD11c (HL3) from BD Pharmingen (USA). We performed cell-surface staining according to standard techniques, and we analysed stained cells using a FACS Canto II cytometer and either CellQuest (BectonDickinson, USA) or FlowJo software (Tree Star, USA).

### Thymus-dependent and -independent antibody production

We used 2,4,6-trinitrophenyl (TNP)-keyhole limpet haemocyanin (KLH) (Biosearch Technologies, USA) and TNP-AminoEthylCarboxyMethyl-FICOLL (Biosearch Technologies) as a thymus-dependent antigen and thymus-independent antigen, respectively. To examine antibody production against thymus-dependent antigens, mice (female C57BL/6J background at 8 weeks of age) were immunized intraperitoneally with 100 μl of 100 μg TNP(29)-KLH (Biosearch Technologies) emulsified with Imject Alum (Thermo Scientific, USA). On day 21, mice were given an intraperitoneal booster injection with the same amount of TNP-KLH/Imject Alum. On days 0, 14 and 30, the serum was collected and TNP-specific IgG1 and IgM were determined by ELISA using 20 μg ml^−1^ TNP(30)-bovine serum albumin (BSA)-coated plates and alkaline phosphatase-conjugated polyclonal rabbit antibodies to mouse IgG1 and IgM (Zymed, 61–0122, 1:1,000 dilution and 62–6822, 1:1,000 dilution).

To elucidate the antibody production against thymus-independent antigens, mice (female C57BL/6J background at 8 weeks of age) were immunized intraperitoneally with 100 μl of 100 μg TNP(65)-AminoEthylCarboxyMethyl-FICOLL (Biosearch Technologies). On days 0 and 7, the serum was collected and TNP-specific IgG3 and IgM were determined by ELISA using 20 μg ml^−1^ TNP(30)-BSA-coated plates and alkaline phosphatase-conjugated polyclonal rabbit antibodies to mouse IgG3 and IgM (Zymed, 1100-04, 1:1,000 dilution and 62–6822, 1:1,000 dilution).

### Titration of protein concentration in urine

Urine was collected from mice (male C57BL/6J background at 1 year of age) and total protein levels were measured using a bicinchoninic acid assay kit (Thermo Scientific).

### Collagen-induced arthritis

On day 0, mice (female C57BL/6J background at 6–8 weeks of age) were immunized intradermally near the base of the tail with 100 μl of 2 mg ml^−1^ chicken IIC (Sigma, USA) emulsified with CFA composed of IFA (Thermo Scientific) plus *M. tuberculosis* H37Ra (Difco, USA; 1.65 or 2.5 mg ml^−1^)^22^. On day 21, mice were given an intradermal booster injection near the previous injection sites with the same amount of IIC/CFA. We judged the development of arthritis in C57BL/6J mice by macroscopic evaluation. Arthritis development in each paw was graded as follows: 0, no change; 1, mild swelling; 2, obvious joint swelling; 3, severe joint swelling and ankylotic changes (maximum of 12 points for individual mice)^56^.

To examine the therapeutic effect of CTRP6 for autoimmune arthritis, we injected rhCTRP6 to arthritic mice. DBA/1J mice (female, 6–8 weeks of age) were immunized with 100 μl of 2 mg ml^−1^ IIC emulsified with CFA (Difco: 0.5 mg ml^−1^ *M. tuberculosis* H37Ra) on day 0 and on day 21 as described above. Then, 300 ng/30 μl rhCTRP6 (Aviscera Bioscience, USA) or control (30 μl PBS or 300 ng/30 μl rhCTRP9: Aviscera Bioscience) was injected every day into the articular cavity of the left (CTRP6) or right (control) knee joint from day 28. We judged the development of arthritis in DBA/1J mice by macroscopic evaluation. Arthritis development in each paw was graded as follows: 0, no change; 1, erythema and mild swelling confined to the tarsal joints; 2, erythema and mild swelling extending from the tarsal joint to digit; 3, erythema and moderate swelling extending from metatarsal joints; 4, erythema and severe swelling encompassing the ankle, foot and digits, or ankylosis of the limb (maximum four points for one leg of individual mice)^57^. WT and *C3*^−/−^ mice (C57BL/6 background, female, 6–8 weeks of age) were immunized with 200 μl of 2 mg ml^−1^ IIC emulsified with CFA composed of IFA (Thermo Scientific) plus *M. tuberculosis* H37Ra (Difco; 0.5 mg ml^−1^) on day 0 and on day 14 as described above. RhCTRP6 (Aviscera Bioscience) or rhCTRP9 (Aviscera Bioscience) (300 ng/30 μl for each) as a control was injected every day into the articular cavity of the left (CTRP6) or right (control) knee joint from day 21. We checked possible aggregation of rhCTRP6 protein by gel filtration chromatography and used the monomer form rhCTRP6, which is free from any aggregation. We judged the development of arthritis by macroscopic evaluation as described above^56^.

### Histopathology

Mice were killed under ether anaesthesia, and their hindlimbs were processed for histopathology. Whole-ankle joints were fixed in 10% formalin in 1 mM phosphate buffer, pH 7.2, decalcified in 10% formic acid and embedded in paraffin. Serial sections of 2–3 μm thickness were taken sagittally through the talus and stained with haematoxylin and eosin for examination by light microscopy. The lesions, including the calcaneus bone and anterior and posterior synovial tissues at the ankle joints, were evaluated histopathologically. Histological severity of each joint was graded as follows: 0, normal; 1, thickening and proliferation of the synovial lining, with slight inflammatory cell infiltration; 2, grade 1 changes plus granulomatous lesions in the synovial sublining tissue; 3, grade 2 changes plus pannus formation and bone destruction. Arthritis index of the ankle joint was estimated from the average grade of talus and around bones including tibia and calcaneum of each mouse.

For immunostaining, limbs were frozen and embedded in special cryomedium SCEM (Leica Microsystems, Japan) and cryosectioned (5 μm thickness) onto a cryofilm (Leica Microsystems). The sections were fixed in cold acetone for 5 min and blocked with 20% goat serum (Wako, Japan)/1% BSA (Sigma) in PBS at room temperature for 1 h. Rat anti-C3 antibody (Abcam, UK, ab11862, 1:1,000 dilution), rat anti-IgG2a (R&D Systems, USA, 344701, 1:1,000 dilution) and Alexa Fluor 488 anti-rat IgG (Invitrogen, USA, A-11006, 1:1,000 dilution) were used for immunostaining. Nuclei were stained with 4′,6-diamidino-2-phenylindole. Relative fluorescence intensity of C3b was determined by ImageJ. The slides were visualized on a Nikon A1Rsi confocal microscope (Nikon, Japan) operated by NIS-Elements software (Nikon).

### IIC-specific antibody titration

For titration of IIC-specific antibodies in CIA, we collected serum samples from mice before primary immunization (pre) and on days 14, 28 and 42 after immunization and diluted them 1:100. We performed ELISA using 20 μg ml^−1^ IIC-coated plates and alkaline phosphatase-conjugated polyclonal rabbit antibodies to mouse IgM and IgG (Zymed, 62–6822, 1:1,000 dilution and 62–6522, 1:1,000 dilution).

### IIC-specific LN cell response

LN cells were harvested from mice at day 7 after IIC immunization. LN cells were cultured in the absence or presence of 100 or 200 μg ml^−1^ denatured IIC for 72 h, followed by incorporation of [^3^H] thymidine (0.25 μCi ml^−1^) (Amersham, UK) for 6 h. Then cells were harvested with the Micro 96 cell harvester (Skatron, Norway), and radioactivity was measured with Micro Beta (Pharmacia Biotech, USA).

### Collagen antibody-induced arthritis (CAIA)

CAIA was induced by injecting a mixture of five mouse monoclonal anti-IIC antibodies together with LPS (Chondrex, USA). Mice (female C57BL/6J background at 8 weeks of age) received an intravenous injection of 5 mg anti-IIC mAb on day 0, and i.p. injection of 50 μg (mild) or 100 μg (severe) LPS on day 3. We judged the development of arthritis by macroscopic evaluation. Arthritis score was determined as described in CIA^58^.

### Titration of C3a and C5a

To measure C3a and C5a levels in plasma, we collected plasma from mice. We performed sandwich ELISA using the capture antibody-coated plates and detection antibodies against C3a and C5a (BD Pharmingen, C3a: BD558250, 1:1,000 dilution and BD558251, 1:1,000 dilution, C5a: BD558027, 1:1,000 dilution and BD558028, 1:1,000 dilution).

### *In vitro* complement activation assay

Plates (Nunc) were coated with the OVA/anti-OVA immune complex (OVA: Sigma, 5 μg ml^−1^ and anti-OVA Ab: Millipore, Germany, AB1225, 1:500,000 dilution), 50 μg ml^−1^ mannan (Sigma) and 200 μg ml^−1^ LPS (Sigma) for assay of complement activation of CP, LP and AP, respectively^59^,^60^,^61^. Diluted mouse serum was incubated on plates at 37 °C for 1 h. We detected the deposition of C3b by rat mAb against mouse C3 (Abcam, ab11862, 1:1,000 dilution). The CP and LP activity was assayed in GVB^++^ buffer, and AP activity was assayed in GVB/Mg^2+^EGTA buffer. For reconstitution experiments, we assayed AP activity using *C1qtnf6*^−/−^ serum in GVB/Mg^2+^EGTA buffer premixed with various concentrations of rhCTRP6 (BioVendor). In addition, we detected the formation of MAC by rabbit polyclonal antibody to mouse MAC (Abcam, ab55811, 1:1,000 dilution). Plates were coated with 50 μg ml^−1^ LPS, and we assayed the AP activity using *C1qtnf6*^−/−^ mice serum in GVB/Mg^2+^EGTA buffer premixed with various concentrations of rhCTRP6.

### C3 convertase activity assay

To elucidate the effect of CTRP6 on AP C3 convertase activity, 1 μg human C3 (Sigma), 1 μg human factor B (CompTech, USA), 100 ng human factor D (CompTech) and 0–100 ng rhCTRP6 (Aviscera Bioscience) in 15 μl of reaction buffer (2 mM CaCl_2_, 0.5 mM MgCl_2_, 20 mM Tris-HCl (pH 7.6)) were incubated at 37 °C for 1 h. The samples were subjected to SDS–PAGE and visualized by CBB staining. The inhibitory effect of CTRP6 on AP C3 convertase activity was calculated from the intensity of factor B protein band, measured using ImageJ. To investigate the association between CTRP6 and factor H, 1 μg C3, 1 μg factor B and 100 ng factor D with/without 100 ng rhCTRP6 and/or 1 μg factor H (CompTech) in 15 μl of reaction buffer were incubated at 37 °C for 1 h, and the reaction mixtures were subjected to SDS–PAGE and visualized by CBB staining.

### Titration of C3 and factor B

C3 and factor B levels in sera were measured using an ELISA kit (C3: Abcam, ab157711 and factor B: USCN Life Science, USA, sE92011Mu).

### Competitive binding assay

For measurement of competition between human CTRP6 and human factor B for binding to C3(H_2_O), a mixture of 20 μg C3(H_2_O); 20 μg factor B (provided by T. Fujita); 0, 20 or 100 μg rhCTRP6 (Aviscera Bioscience); 1.5 μg anti-C3 antibody (Abcam, ab11871); and 20 μl Protein A/G PLUS-Agarose (Santa Cruz) in 0.5 ml of a reaction buffer (2 mM CaCl2, 0.5 mM MgCl2, 20 mM Tris-HCl (pH 7.6)) was incubated at 37 °C for 2 h. After washing with PBS/0.05% Tween-20, samples were subjected to SDS–PAGE and visualized by CBB staining. Immunoprecipitated factor B was semi-quantified by the band intensity of factor B, which is normalized to the band intensity of C3β using ImageJ.

### Immunoprecipitation and western blotting

We collected *C1qtnf6*^−/−^ mouse serum and pre-cleared with Protein A/G Plus-Agarose (Santa Cruz). To immunoprecipitate CTRP6-associated proteins, 200 ng CTRP6 (BioVendor) or 10 mM acetic acid (control) was added to 1 ml pre-cleared *C1qtnf6*^−/−^ mouse serum, and immunoprecipitated with 2 μg rabbit polyclonal antibody (Anaspec, 54561) to CTRP6. Immunoprecipitates were washed five times with PBS-1% Tween-20 and dissolved in SDS–PAGE running buffer (0.04 M Tris-HCl; 0.022 M glycine; 1% (w/v) SDS; pH 8.3). Aliquots of immunoprecipitate were separated by SDS–PAGE on 12.5% gels. Resolved proteins were transferred onto polyvinylidene difluoride membranes. Membranes were blocked with 5% skim milk/Tris-buffered saline containing 0.1% Tween-20 (TBST) at room temperature for 1 h, followed by incubation overnight at 4 °C with rat mAb against C3 (Abcam, ab11862, 1:1,000 dilution) or rabbit polyclonal antibody against CTRP6 (Anaspec, 54562, 1:1,000 dilution). The membranes were then washed three times with TBST, followed by 1 h incubation at room temperature with HRP-conjugated goat polyclonal antibody against rat IgG (Zymed, 81–9520, 1:1,000 dilution). The membranes were washed six times with TBST and visualized using the ECL Prime Western Blotting Detection System (GE Healthcare, Japan).

### SPR-based interaction assay

Each human recombinant protein (CTRP6, Aviscera Bioscience; C3b, Alpha Diagnostic) were linked to CM5 chips via amino coupling. The coupling was performed according to the standard *N*-ethyl-*N*′-(3-dimethyl aminopropyl)-carbodiimide hydrochloride/*N*-hydroxysuccinimide procedure. In brief, the CM5 matrix was activated by injection of 70 μl of a 1:1 mixture of 400 mM *N*-ethyl-*N*′-(3-dimethyl aminopropyl)-carbodiimide hydrochloride and 100 mM *N*-hydroxysuccinimide at a flow rate of 20 μl min^−1^, followed by application of the appropriate recombinant protein solution. Activated functional groups on the sensor chip not saturated by proteins were blocked with 1 M ethanolamine. Binding of human CTRP6 was stopped when the desired level of about 100 resonance units was reached. Next, the indicated analyte (CTRP6, factor H, Sigma; C3(H_2_O), provided by T. Fujita) was injected. C3(H_2_O) was generated by five freeze/thawing cycles of C3 (ref. 62). Regeneration of the sensor chip was achieved by injecting the buffer described below for 3 min. The SPR assay and kinetics analysis was performed using a BIAcore 2000. The experiments were routinely performed in 10 mM HEPES (pH 7.4) buffer containing 150 mM NaCl, 3 mM EDTA and 0.005% surfactant P20 at a flow rate of 20 μl min^−1^.

### Reverse passive Arthus (RPA) reaction

For IgG-mediated cutaneous RPA reaction, mice were intradermally injected with 10 μg anti-OVA Ab (Millipore, AB1225), and then intravenously with 500 μg OVA/1% Evans Blue (OVA; Sigma and Evans Blue; Wako) in PBS (pH 7.4). Skin was harvested 4 h later and incubated with dimethylformamide; eluted Evans blue in the supernatants was quantified by measuring the absorbance at 570 nm (ref. 63).

### Experimental autoimmune encephalomyelitis (EAE)

The MOG_35–55_ peptide (MEVGWYRSPFSRVVHLYRNGK) was synthesized and purified by HPLC at our institute (Dr S. Imajoh-Ohmi, Division of Medical Proteomics Laboratory, Institute of Medical Science, University of Tokyo). Mice were immunized subcutaneously in flanks on day 0 with 300 μg MOG_35–55_ peptide emulsified in CFA (1:1), which consisted of IFA with 5 mg ml^−1^ *M. tuberculosis* (Difco). On day 7, mice were given a booster injection subcutaneously in flanks with the same amount of MOG/CFA. We judged the development of the severity of EAE by macroscopic evaluation. The severity score was graded as follows: 0, no change; 0.5, partially limp tail; 1, paralysed tail; 2, hind limp paresis; 2.5, one hindlimb paralysed; 3, both hindlimbs paralysed; 3.5, hindlimbs paralysed and weakness in forelimbs; 4, forelimbs paralysed^64^.

### Gel filtration chromatography

C57BL/6 mouse serum (100 μl) or 1 μg/100 μl rhCTRP6 (Aviscera Bioscience) were fractionated through a 16/60 Sephacryl S-300 (GE Healthcare, USA) column (1.6 × 60 cm) in PBS at flow rate of 0.5 ml min^−1^. The fractionated samples were subjected to ELISA and the protein concentration was measured with a spectrophotometer. Thyroglobulin (669 kDa, Sigma), catalase (232 kDa, Sigma), BSA (66 kDa, Sigma) and OVA (45 kDa, Sigma) were used as molecular-weight makers.

### Primary fibroblast-like synoviocytes

The primary synoviocytes were harvested from synovium of the knee and ankle and were cultured in DMEM medium (Gibco, USA) containing 10% fetal bovine serum and 1% penicillin–streptomycin. Cells were stimulated with IL-1α (0, 0.1, 1 and 10 ng ml^−1^) for 24 h. *C1qtnf6* expression was measured by semi-quantitative PCR analysis.

### BM-derived macrophages

To generate BM-derived macrophages, BM cells at 2 × 10^5^ cells per millilitre were cultured in a 100-mm untreated dish using RPMI 1640 medium supplemented with 10% fetal bovine serum and 20 ng ml^−1^ recombinant macrophage colony-stimulating factor (PeproTech, USA). On day 3, we added another 10 ml of this medium to the dish. At day 7, we collected BM-derived macrophages and the cells at 1 × 10^5^ cells per millilitre in a 96-well dish were stimulated with rhCTRP6 for 24 h. IL-10 levels in the culture supernatants were measured using Mouse IL-10 ELISA MAX Standard (Biolegend).

### Samples from RA patients and healthy controls

Serum samples were collected from 30 Japanese RA patients diagnosed by rheumatologists according to the criteria of the American College of Rheumatology (ACR) in 1987 (ref. 65) or the 2010 ACR/EULAR classification criteria^66^. The mean age of the RA patients was 56.6 years; 20.0% were males. Serum samples were also collected from 22 healthy control subjects (mean age, 27.8 years; 18.2%, males). Frozen sections of synovium were fixed in 4% paraformaldehyde at room temperature for 15 min and blocked with 1% BSA in PBS for 30 min. For immunostaining of CTRP6, a rabbit anti-CTRP6 antibody (Abcam, ab36900, 1:100 dilution) and anti-rabbit IgG (Bio-Rad, 172–1019, 1:50 dilution) were used, and a rabbit IgG (Dako, Denmark, X0903, 1:100 dilution) was used as a control. Antibody binding was visualized using diaminobenzidine tetrahydrochloride (Nichirei Bioscience, Japan). For fluorescent immunostaining, a rabbit anti-CTRP6 antibody (Abcam, ab36900, 1:100 dilution), rabbit IgG (Bio-Rad, 172–1019, 1:50 dilution), mouse anti-CD3 antibody (Biolegend, 344802, 1:50 dilution), mouse anti-CD68 antibody (Biolegend, 556059, 1:50 dilution), mouse anti-Hsp47 antibody (Abcam, ab77609, 1:50 dilution), mouse IgG2b (BD Pharmingen, 557351, 1:50 dilution), mouse anti-CD20 antibody (Biolegend, 302301, 1:50 dilution), mouse IgG1 (Biolegend, 401401, 1:50 dilution), Alexa Fluor 488 anti-rabbit IgG (Invitrogen, A11034, 1:50 dilution) and Alexa Fluor 546 anti-mouse IgG (Invitrogen, A11030, 1:50 dilution) were used. Nuclei were stained with 4′,6-diamidino-2-phenylindole (Vector Labs, USA). The slides were visualized on a fluorescence microscope (Keyence, Japan). All the samples were collected from patients from whom we obtained informed consent at the University of Tsukuba Hospital. This study was reviewed and approved by the ethics committee of the University of Tsukuba.

### Statistical analysis

Incidence of CIA, CAIA and EAE was evaluated by the *χ*^2^-test, and the severity score by the Mann–Whitney *U*-test. Student's *t*-test was used for all other statistical evaluations.

## Additional information

**How to cite this article:** Murayama, M. A. *et al*. CTRP6 is an endogenous complement regulator that can effectively treat induced arthritis. *Nat. Commun.* 6:8483 doi: 10.1038/ncomms9483 (2015).

## Supplementary Material

Supplementary Information Supplementary Figures 1-10, Supplementary Tables 1-3, Supplementary Methods and Supplementary References
