# Supplementary material for: CTRP6 is an endogenous complement regulator that can effectively treat induced arthritis
Source: Nat Commun. 2015 Sep 25;6:8483. doi: 10.1038/ncomms9483 (PMC4598845; doi:10.1038/ncomms9483)
Supplement: Supplementary Information — Supplementary Figures 1-10, Supplementary Tables 1-3, Supplementary Methods and Supplementary References [file ncomms9483-s1.pdf]

Supplementary Information
Supplementary Figures

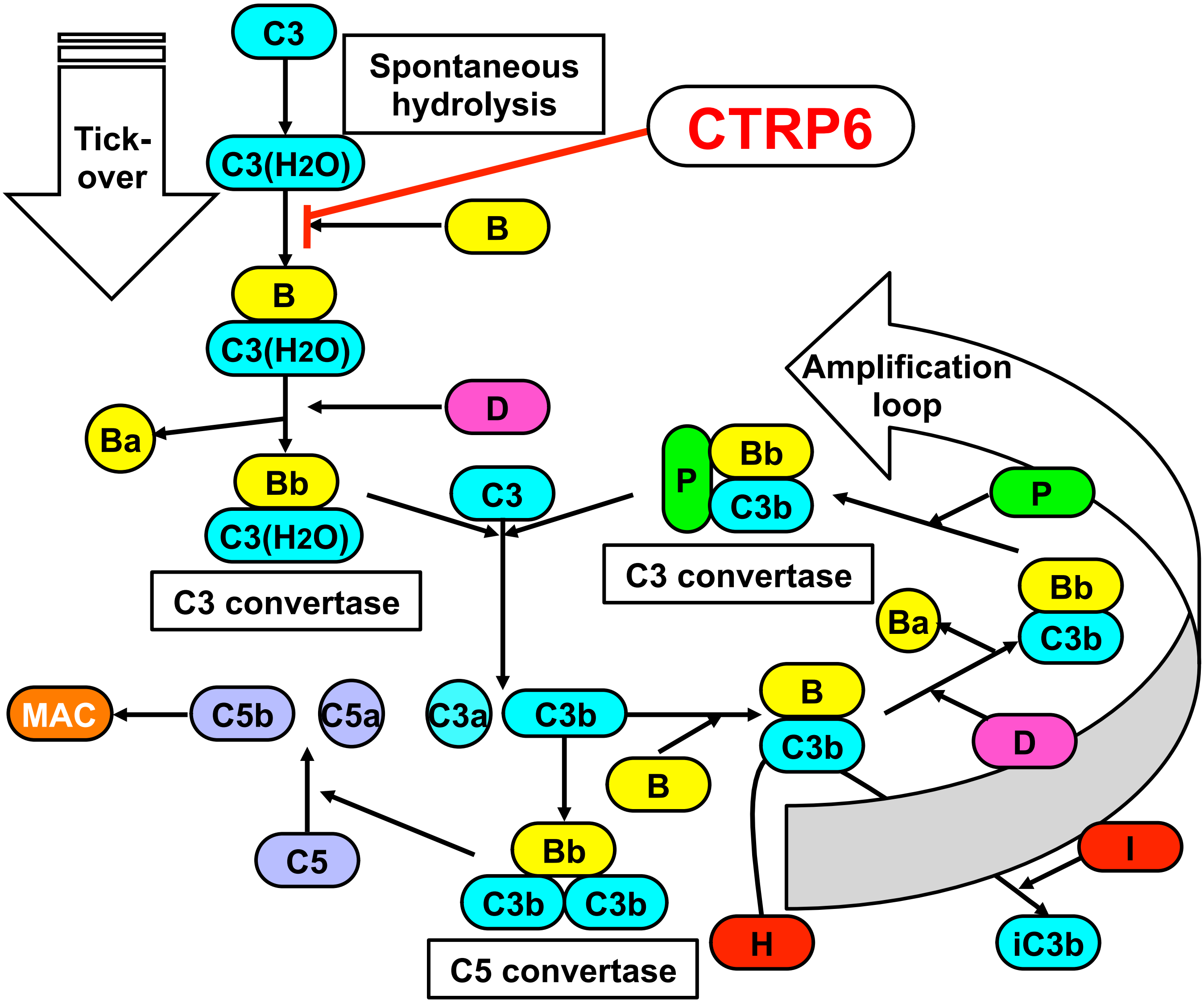

Supplementary Figure 1. A possible regulatory mechanism for complement activation by CTRP6. C3 is spontaneously hydrolyzed to C3(H_2O) at low levels (C3 tick-over), and factor B (B) binds C3(H_2O). C3(H_2O)B is activated by factor D (D) to generate C3 convertase (C3(H_2O)Bb). C3(H_2O)Bb activates C3 into C3a (anaphylatoxin) and C3b. Also, B binds C3 to generate C3bBb. C3bBb is stabilized in the presence of factor P (P) and forms C3bBb-P (C3 convertase) (amplification loop). C3bBb also binds another C3b to generate C5 convertase (C3bBbC3b). C5 convertase activates C5 into C5a (anaphylatoxin) and C5b, resulting in the formation of MAC. CTRP6 inhibits the binding C3 and B, which is the initial step of alternative pathway activation, causing the suppression of C3a and C5a generation and MAC formation.

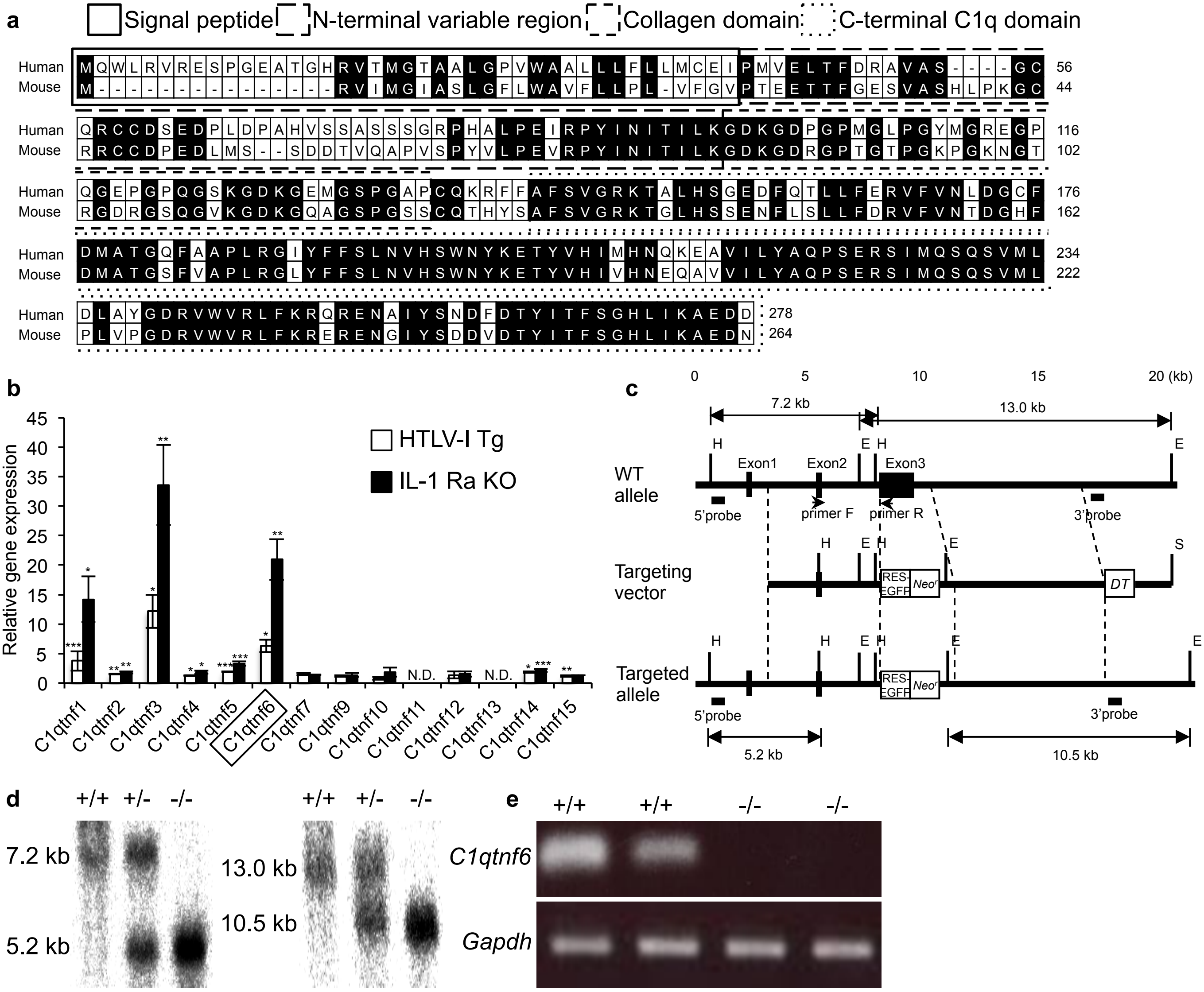

Supplementary Figure 2. Generation of *C1qtnf6*$^{-/-}$ mice.
(**a**) ClustalW alignment of the human and mouse CTRP6 amino acid sequences. The Gene ID of human and mouse *C1qtnf6* are 114904 and 72709. Identical amino acids are shown as white text on black background. The amino acid identity of C1q domain is 82 %. (**b**) The relative expression of *C1qtnf* family mRNA in the joints of HTLV-I Tg and mice IL-1 Ra KO mice to WT mice were determined by quantitative RT-PCR (WT: n = 6, HTLV-I Tg: n = 6, and IL-1 Ra KO: n = 7). *C1qtnf8* is not conserved in mice. Average and s.e.m. are shown. *$P < 0.05$, **$P < 0.01$, and ***$P < 0.001$. Student's *t*-test. (**c**) Structure of the mouse *C1qtnf6* locus (WT allele), the *C1qtnf6* targeting construct (Targeting vector), and the predicted mutated *C1qtnf6* gene (Mutant allele). Exons are represented by black boxes. Exon 3 of the *C1qtnf6* gene, encoding the C1q domain, was replaced by the neomycin resistance gene (*Neo*^{r}). For negative selection, a diphtheria toxin gene (*DT*) was attached to the 3' end of the genomic fragment. *Sac*II was used for linearization. (**d**) The *C1qtnf6*-deficient allele was confirmed in the *Hind*III (H)-digested genome by Southern blot hybridization analysis using the 5' probe (**left**). The *C1qtnf6*-deficient allele was confirmed in the *Eco*RV (E)-digested genome by Southern blot hybridization analyses using the 3' probe (**right**). (**e**) Lack of *C1qtnf6* expression in the spleen was confirmed by RT-PCR using primers F and R (**Supplementary Table 1**).

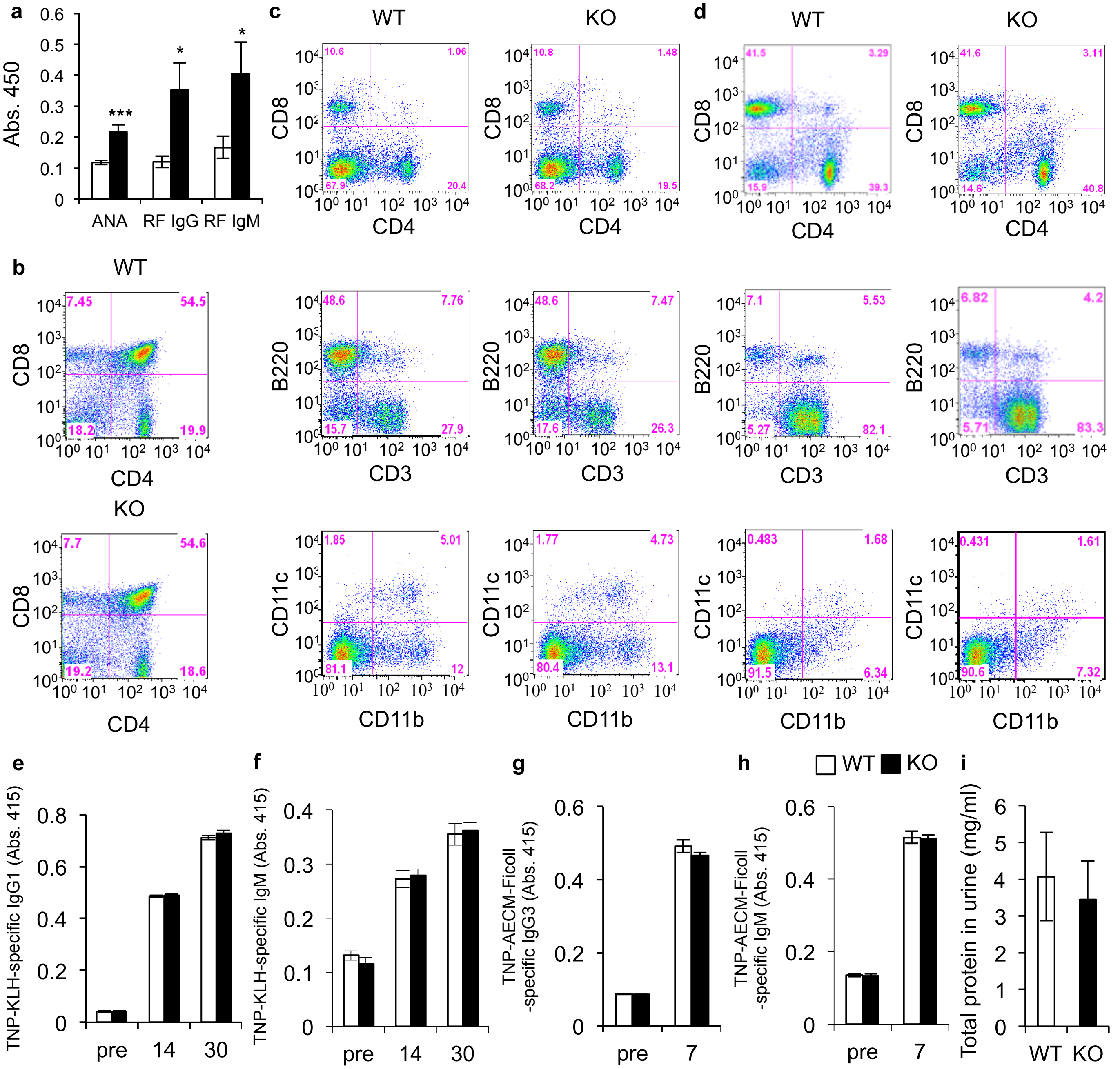

Supplementary Figure 3. lymphocyte population, antibody production and renal function in *C1qtnf6*$^{-/-}$ mice.

(**a**) The sera of WT and *C1qtnf6*$^{-/-}$ mice (KO) (1 year of age) were collected and ANA, RF IgG and RF IgM levels were determined by ELISA (WT: n = 15 and KO: n = 20). **P* < 0.05 and ****P* < 0.001. Student's *t*-test. The expression of CD4, CD8, CD3, B220, CD11b, and CD11c in thymocytes (**b**), splenocytes (**c**) and LN cells (**d**) in WT and *C1qtnf6*$^{-/-}$ (KO) mice were analyzed by flow cytometry. The numbers in each panel indicate the percentage of cells within the specific gate as a fraction of total cells. These data are representative of six mice. (**e-h**) The sera before (pre) and after 7, 14, and 30 day immunization were collected from WT and *C1qtnf6*$^{-/-}$ (KO) mice. TNP-KLH–specific IgG1 (**e**) and IgM (**f**), TNP-AECM-Ficoll–specific IgG3 (**g**), and IgM (**h**) levels were determined by ELISA. The data from two independent experiments were combined (n = 10 each in e, and f, and n = 9 in g and h). Student's *t*-test. (**i**) Total protein in the urine from aged WT and *C1qtnf6*$^{-/-}$ mice was measured by BCA protein assay (WT: n = 10 and KO: n = 9). Student's *t*-test. Average and s.e.m. are shown.

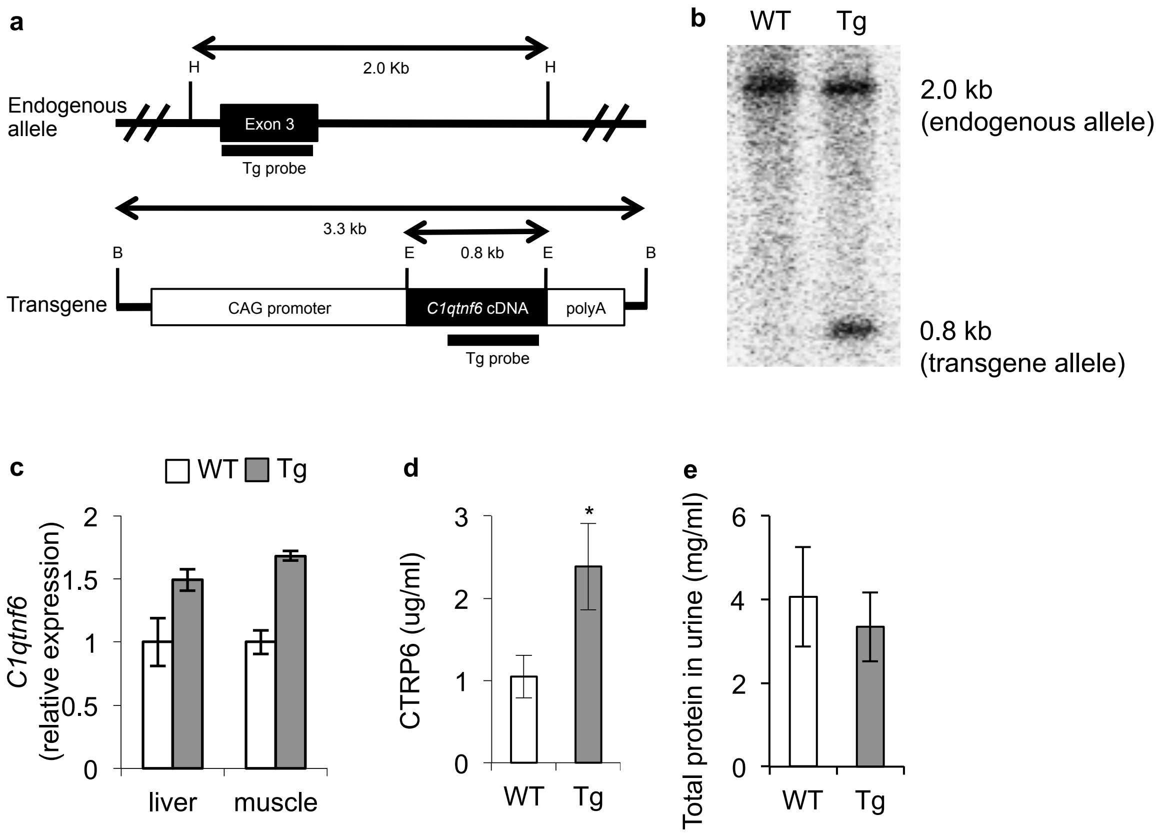

Supplementary Figure 4. Generation of *C1qtnf6* Tg mice.
(**a**) The structure of the mouse *C1qtnf6* locus (endogenous allele) and CAG-*C1qtnf6*-polyA fragment (transgene). The *C1qtnf6* cDNA was inserted into the pCXN2 plasmid, a derivative of pCAGGS containing the CAG promoter (1700 bp) and rabbit beta-globin polyA (534 bp), at the *Eco*RI (E) site downstream of the CAG promoter. The CAG promoter (1700 bp) is composed of the cytomegalovirus (CMV) immediate-early enhancer (279 bp), chicken beta-actin promoter (279 bp), and chicken beta-actin intron (963 bp). The *Bam* HI (B)–*Pvu*I fragment containing the CAG-*C1qtnf6*-polyA was microinjected into the pronuclei of fertilized C57BL/6J mouse eggs. (**b**) The transgene was detected by Southern blot hybridization after digestion of DNA from mouse tails with *Hind*III (H) and *Eco*RI (E). (**c**) The *C1qtnf6* transcripts in the liver and muscle were assessed by real-time PCR (n = 3 each). Student's *t*-test. (**d**) The CTRP6 concentration in serum was assessed by ELISA (n = 8 each). Student's *t*-test. (**e**) Total protein in the urine from aged WT and *C1qtnf6* Tg (Tg) mice was measured by BCA protein assay (WT: n = 10 and Tg: n = 10). *$P < 0.05$. Student's *t*-test. Average and s.e.m. are shown. Similar results were obtained in another independent experiment.

Supplementary Figure 5.

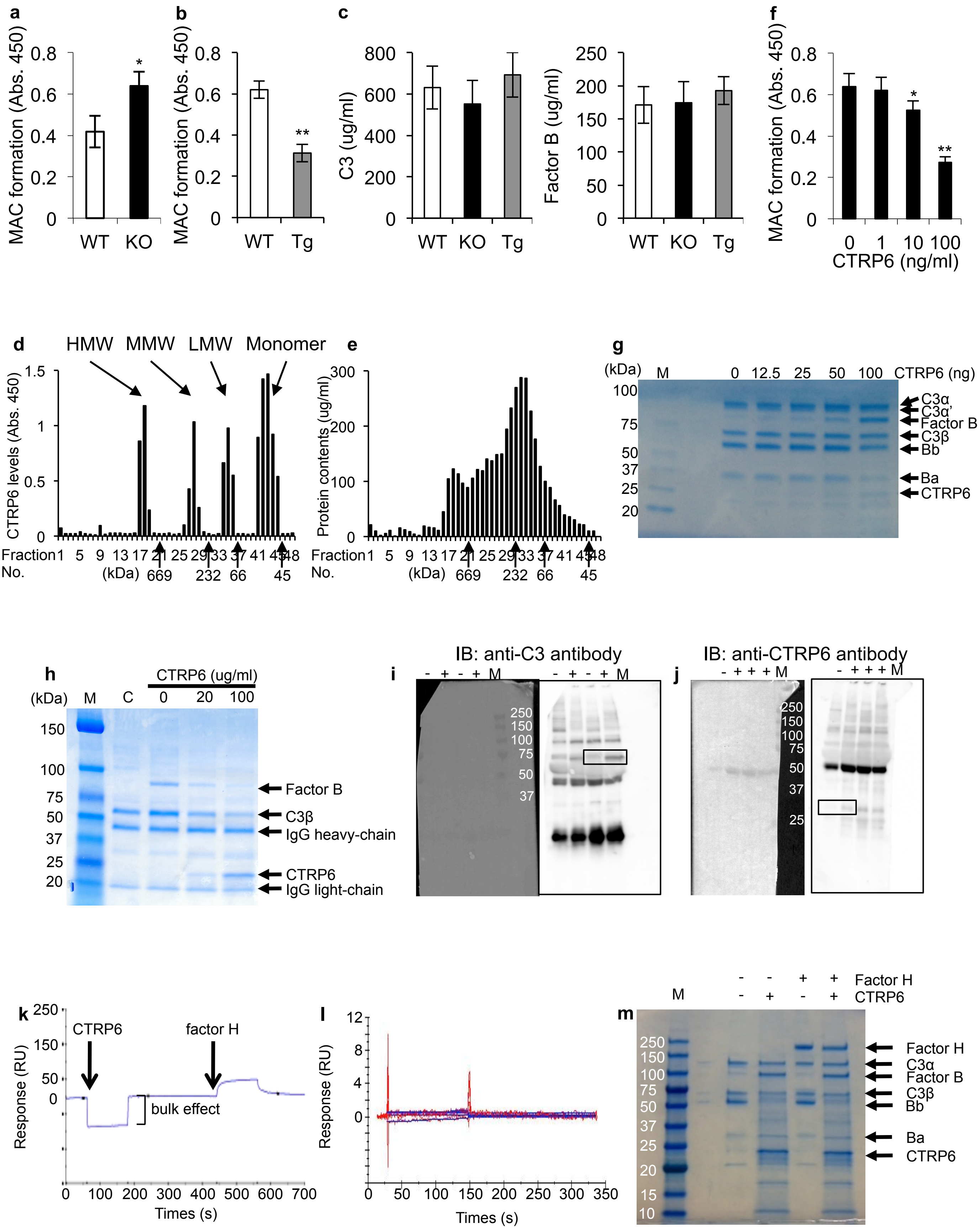

Supplementary Figure 5. CTRP6 inhibits C3b deposition and MAC formation via regulation of AP activation.

(**a**) The complement AP activation in 10% WT and *C1qtnf6*$^{-/-}$ mouse serum was measured by MAC formation (WT, KO: n = 8 each). **P* < 0.05. Student's *t*-test. (**b**) The complement AP activation in 10% WT and *C1qtnf6* Tg mouse serum was measured by MAC formation (WT, Tg: n = 5 each). **P* < 0.01. Student's *t*-test. (**c**) C3 (**right**) and factor B (**left**) concentrations in the serum from WT mice, *C1qtnf6*$^{-/-}$ mice (KO) and *C1qtnf6* Tg mice (Tg) were measured by ELISA. (WT, KO and Tg: n = 8 each). Student's *t*-test. (**d**, **e**) WT mouse serum was chromatographed on a Sephacryl S-300 column. (**d**) CTRP6 concentrations were measured by ELISA. Upper arrows show elution positions of monomer, low-, middle- and high-molecular weight (LMW, MMW and HMW) form of CTRP6. (**e**) Total protein concentration was monitored with a spectrophotometer (Abs. 280). (**f**) *C1qtnf6*$^{-/-}$ mouse serum (10%) was activated by LPS in the presence of rhCTRP6, and MAC formation were evaluated (n = 8). **P* < 0.05 and ***P* < 0.01. Student's *t*-test. (**g**) Full-size image of **Figure 4d**. The lane of molecular mass maker (kDa) is represent as '**M**'. (**h**) Full-size image of **Figure 4e** with molecular mass maker (**M**). C3(H_2O) was immunoprecipitated with anti-C3 antibody as a control in lane '**C**'. (**i, j**) Full-size scans of Western blots (**right**) and transferred membrane (**left**) of **Figure 4f**. (**k, l**) Surface plasmon resonance analysis of CTRP6 binding to C3b and factor H. (**k**) RhCTRP6 and factor H (**H; positive control**) were flowed over a C3b-immobilized sensor chip. (**l**) Factor H was flowed over a rhCTRP6-immobilized sensor chip. (**m**) The inhibition of AP C3 convertase activity by CTRP6 did not relate factor H. A reaction mixture of C3, factor B and factor D with/without rhCTRP6 and/or factor H was subjected to SDS-PAGE and visualized by CBB staining. All the data were reproduced in another independent experiment with similar results. Average and s.e.m. are shown.

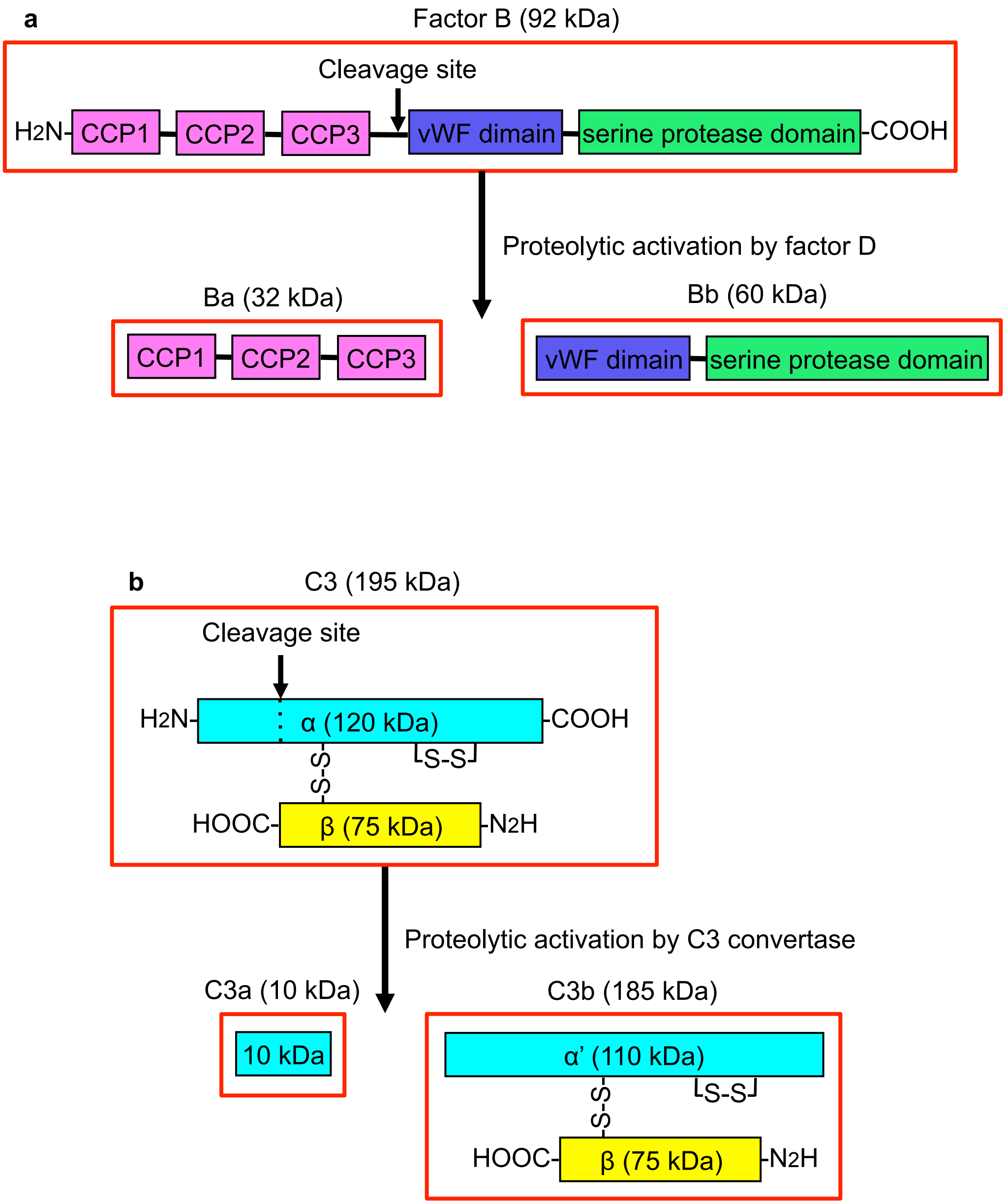

Supplementary Figure 6. Factor B and C3 processing.
(**a**) Factor B is a zymogen that contains three N-terminal complement control protein (CCP) domains, a von Willebrand factor A (vWF) domain, and a C-terminal serine protease (SP) domain. B (92 kDa) is cleaved to Ba (CCP1–3: 32 kDa) and Bb (vWF and SP domain: 60kDa) by factor D (D). C3- and C3b-bound B are activated by D into C3 convertase and C5 convertase, respectively. (**b**) C3 (195 kDa) is composed of two subunits, C3α (120 kDa) and C3β (75 kDa), linked by a disulfide bond. C3 is activated by C3 convertase, which cleaves C3α into C3a (10 kDa) and C3α' (110 kDa), resulting in the formation of C3b (185 kDa) that is composed of C3α' and C3β (75 kDa).

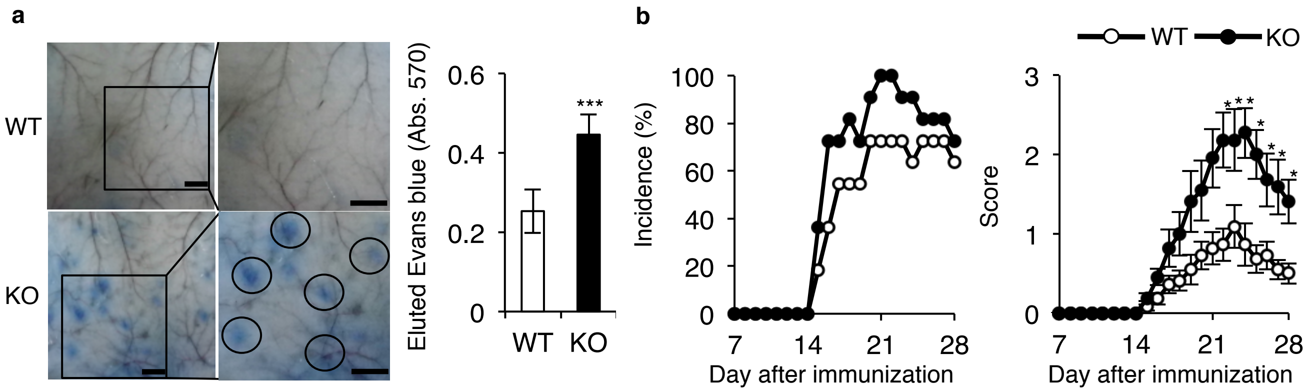

Supplementary Figure 7. The effects of CTRP6 deficiency on the reverse passive Arthus (RPA) reaction and EAE.

(**a**) The dorsal skin of mice after the cutaneous RPA reaction. The circles indicate exuded Evans blue dye. Scale bar, 0.5 cm. These data are representative of six mice. For quantitative analysis of vascular permeability, extracted Evans blue dye was quantitated (WT, KO: n = 6 each). ***$P < 0.001$. Student's *t*-test. Similar result was obtained in another independent experiment. (**b**) Incidence (left) and severity score (right) of EAE (WT: n = 11, KO: n = 11). *$P < 0.05$. χ^2 test and Mann-Whitney *U*-test. All data were reproduced in another independent experiment with similar results. Average and s.e.m. are shown.

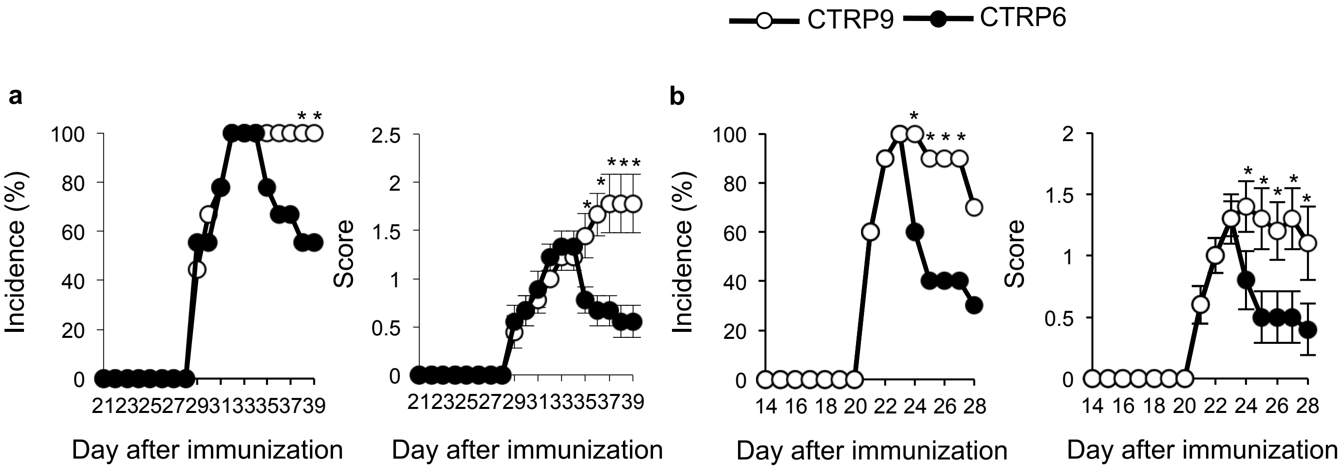

Supplementary Figure 8. Treatment of CIA with CTRP6 and CTRP9.

(**a**, **b**) Incidence and severity score of CIA. (**a**) RhCTRP6 (300 ng), or rhCTRP9 as a control (300 ng), was injected daily into the left or right knee joints of CIA-induced DBA/1J mice from day 34 after primary immunization (n = 9). Similar result was obtained in another independent experiment. *$P < 0.05$. χ^2 test and Mann-Whitney *U*-test. (**b**) RhCTRP6 (300 ng) or rhCTRP9 (300 ng) was injected daily into the left or right knee joints of CIA-induced C57BL/6J mice from day 21 after primary immunization. These data were combined from two independents (n = 10). *$P < 0.05$. χ^2 test and Mann-Whitney *U*-test. Average and s.e.m. are shown.

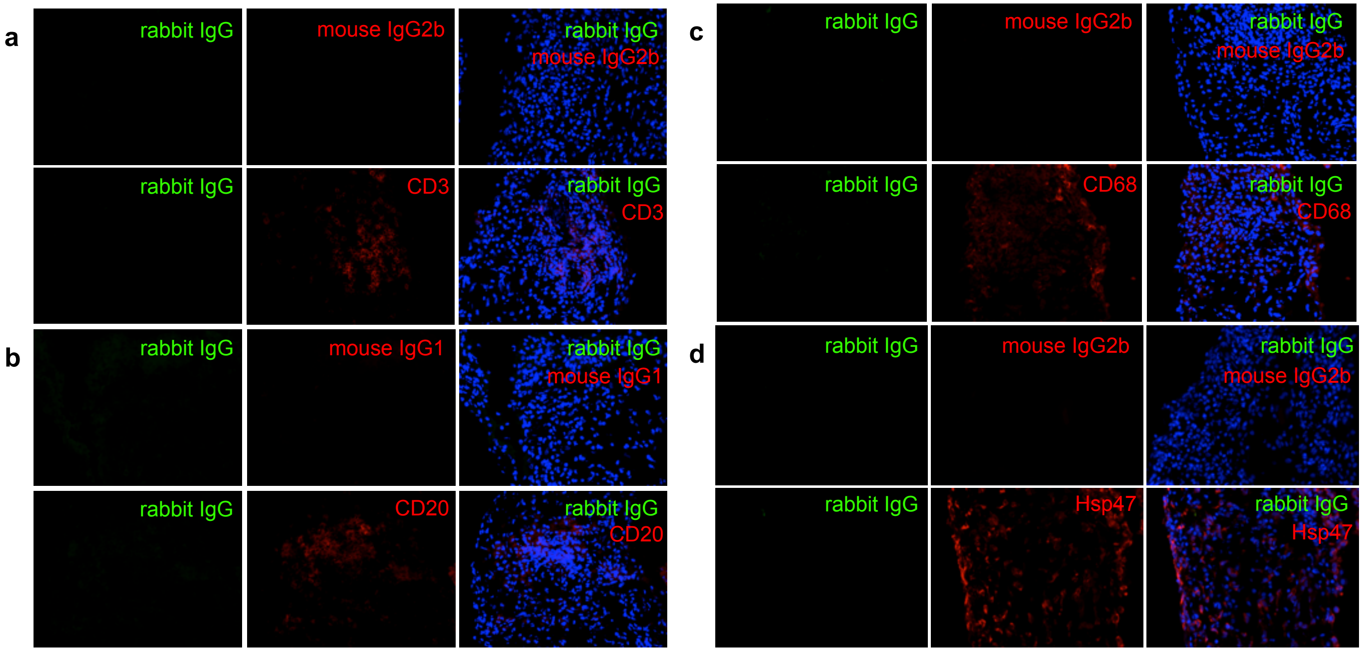

Supplementary Figure 9. Fluorescent immunostaining analysis, related to figure 6.
Cryostat sections of synovium from RA patient was stained with rabbit IgG (isotype of anti-CTRP6 antibody), and anti-CD3 antibody or isotype (**c**), anti-CD20 antibody or isotype (**d**), anti-CD68 antibody or isotype (**e**), anti-Hsp47 antibody or isotype (**f**), and DAPI (nucleus).

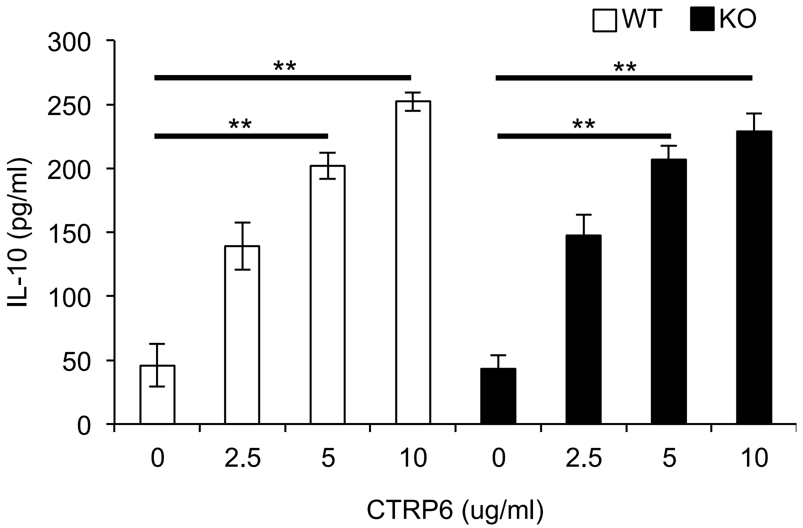

Supplementary Figure 10. CTRP6 regulates IL-10 production from macrophages.
IL-10 production from bone marrow-derived macrophages after rhCTRP6 (0-10 ug/ml) stimulation was measured by ELISA (WT, KO: n = 3 each). Average and s.e.m. are shown. **$P < 0.01$. Student's *t*-test.

Supplementary Tables

Gene name	Forward primer	Reverse primer
C1qtnf1	CCTTGTGCCACGAGTTCAGG	CGCCTTTCAGGATGGTGATG
C1qtnf2	ACTCTTGGCCTGTGCCCTTC	CCCATTCTTCCCACCACTCC
C1qtnf3	CTTCAGCATGTACAGCTATG	GTTGCCCATTCTTAGCCAGACT
C1qtnf4	GACGCTGTCGGTGAAGCTGA	CTTGCCGTGGTTGCTGTAGG
C1qtnf5	GGGCCATTACGACCCCACTA	GTAATCACCCACGCCCACCT
C1qtnf6	AGTCAGGCCGTACATCAACA	ACCTTTGACACCCTGAGAGC
C1qtnf7	CTCGGGCCAATCAGGCTAAG	GTGCCCTTTTCCCCCTTCTC
C1qtnf9	AGGGGCAGAAGGGGGATAAA	CGCCTTTCCAGCCTCTCATT
C1qtnf10	CGAAGGCTACGAGGTGCTCA	GCTGTTGCTGGCGTAGTCGT
C1qtnf11	TGAAGTGTTGCGCTTTGACG	GATCCGCATCCTGAGCAATG
C1qtnf12	GCTGGTGGTTGAGGCCTTCT	GCAGAGACTGGGGCTGTGAA
C1qtnf13	GTCCCTGCCCACTTTCATCC	GCCAGCGTAAAAGGCGATCT
C1qtnf14	CAGCACGGCCACCTATACCA	TTGCAGAGGTCTGCCCACAT
C1qtnf15	CGGACCTGTCCCCAAATCAG	AGGGTGGGGTGTTTCCACCT
Gapdh	TTCACCACCATGGAGAAGGC	GGCATGGACTGTGGTCATGA
Il1b	CAACCAACAAGTGATATTCTCCATG	GATCCACACTCTCCAGCTGCA
Il10	GCTCTTACTGACTGGCATGAG	CGCAGCTCTAGGAGCATGTG
Tnf	GCCTCCCTCTCATCAGTTCT	CACTTGGTGGTTTGCTACGA
F4/80	CCCAGGAGTGGAATGTCAAG	GCTCTCCCCAGGATATTGGT

Supplementary Table 1. The real-time PCR primer sets.

Primer name	Forward primer	Reverse primer
primer set 1	TCCCCGCGGCTCCAAACCATGCTGACTCT	CGTCTAGAAGCACCTAACTGCATGCTGG
primer set 2	CCATCGATATCTGGGCAAGTCCCCATGTCT	ACGCGTCGACTCCTCCTGGGTCATTCTGCA
primer set 3	CTCTTTCTGGCAAGCACATAGCTC	AGAGGAACCCAAGCTTCTTACAGG
primer set 4	AACCTGACTTATGTGTGGGCAG	CAGGATTGTATGAGTGTCTGGG
primer set 5	AGAGGATGTGTGCGTAGTCCAA	TGGATGGACAGATGGATGGATG
primer set 6	TTGAATTCGCAGGATGAGGGTCATCATGG	CGGAATTCAGTTGTCCTCTGCCTTGATC
primer set 7	ACGTGCTGGTTGTTGTGCTGTCTC	CTTTATAGCCACCTTTGTTCATGGC
primer set 8	CTACAGGAACACCAGGGAAGCCA	TTGATCAGGTGGCCACTGAAGGT

Supplementary Table 2. The primer sets.

Primer name	Sequence
WT sense primer 1	GGCATCTCTGGTGCTTACAACCAAG
mutant sense primer 1	AGTTATACGCGTTCGCTCGGTACCCA
common antisense primer 1	GACAGCAAGCTGATCATCCACACTCA
WT sense primer 2	TGGCCTCAGTTTCACTTCTGCAGA
mutant sense primer 2	ATCCTAAAGGGTGACAAAGGGGAC
common antisense primer 2	CTGTGTACGTTGAGGCTGAAGAAG
WT sense primer 3	GTTGATTCTTGCATCTCACCTG
mutant sense primer 3	GCTCGGTACCCATCAAGCTTAT
common antisense primer 3	GATGCAGAGCAATATCACACAG

Supplementary Table 3. The genotyping PCR primers.
